# Synthesis and crystal structures of three organoplatinum(II) complexes bearing natural aryl­olefin and quinoline derivatives

**DOI:** 10.1107/S2056989024004572

**Published:** 2024-05-21

**Authors:** Nguyen Thi Thanh Chi, Pham Van Thong, Nguyen Manh Thang, Pham Ngoc Thao, Luc Van Meervelt

**Affiliations:** a Department of Chemistry, Hanoi National University of Education, 136 Xuan Thuy, Cau Giay, Hanoi, Vietnam; b R&D Center, Vietnam Education and Technology Transfer JSC, Hanoi, Vietnam; c Bac Giang Upper Secondary School for the Gifted, Bac Giang, Vietnam; d University of Engineering and Technology, Vietnam National University, 144 Xuan Thuy, Cau Giay, Hanoi, Vietnam; e Department of Chemistry, KU Leuven, Biomolecular Architecture, Celestijnenlaan 200F, Leuven (Heverlee), B-3001, Belgium; University of Kentucky, USA

**Keywords:** crystal structure, platinum(II) complex, quinoline, aryl­olefin, square-planar coordination

## Abstract

Three organoplatinum(II) complexes bearing natural aryl­olefin and quinoline derivatives, namely, [4-meth­oxy-5-(2-meth­oxy-2-oxoeth­oxy)-2-(prop-2-en-1-yl)phen­yl](quinolin-8-olato)platinum(II), [Pt(C_13_H_15_O_4_)(C_9_H_6_NO)], (**I**), [4-meth­oxy-5-(2-oxo-2-propoxyeth­oxy)-2-(prop-2-en-1-yl)phen­yl](quinoline-2-carboxy­l­ato)platinum(II), [Pt(C_15_H_19_O_4_)(C_10_H_6_NO_2_)], (**II**), and chlorido­[4-meth­oxy-5-(2-oxo-2-propoxyeth­oxy)-2-(prop-2-en-1-yl)phen­yl](quinoline)­plat­inum(II), [Pt(C_15_H_19_O_4_)Cl(C_9_H_7_N)], (**III**), were synthesized and structurally characterized by IR and ^1^H NMR spectroscopy, and by single-crystal X-ray diffraction. The results showed that the cyclo­platinated aryl­olefin coordinates with Pt^II^ *via* the carbon atom of the phenyl ring and the C=C_olefinic_ group. The deprotonated 8-hy­droxy­quinoline (C_9_H_6_NO) and quinoline-2-carb­oxy­lic acid (C_10_H_6_NO_2_) coordinate with the Pt^II^ atom *via* the N and O atoms in complexes (**I**) and (**II**) while the quinoline (C_9_H_7_N) coordinates *via* the N atom in (**III**). Moreover, the coordinating N atom in complexes (**I**)–(**III**) is in the *cis* position compared to the C=C_olefinic_ group. The crystal packing is characterized by C—H⋯π, C—H⋯O [for (**II**) and (**III**)], C—H⋯Cl [for (**III**) and π–π [for (**I**)] inter­actions.

## Abstract 2

The synthesis and crystal structures of three organoplatinum(II) complexes bearing natural aryl­olefin and quinoline derivatives are reported.

## 1. Chemical context

In cancer chemotherapy, three generations of platinum-based drugs, namely cisplatin, carboplatin and oxaliplatin, have been approved all over the world. In addition, some other platinum-based drugs are used in Asia, such as Japan (nedaplatin), China (lobaplatin) and Korea (hepta­platin) (Johnstone *et al.*, 2016 ▸). However, these drugs cause several undesirable side effects and are not universally effective in all types of human cancer. Recently, many organoplatinum(II) complexes possessing natural aryl­olefin ligands and either amine or N-heterocyclic carbene have been synthesized with the aim of minimizing toxicity and diversifying hopeful anti-cancer agents. The tested cytotoxicity results show that many of them exhibit higher activity than cisplatin on some human cancer cell lines such as KB, Lu-1, Hep G2 and MCF-7 (Da *et al.*, 2012 ▸, 2015 ▸; Thi Hong Hai *et al.*, 2019 ▸; Nguyen Thi Thanh *et al.*, 2017 ▸; Chi *et al.*, 2018 ▸, 2020 ▸; Van Thong *et al.*, 2022 ▸).

**Figure 1:**
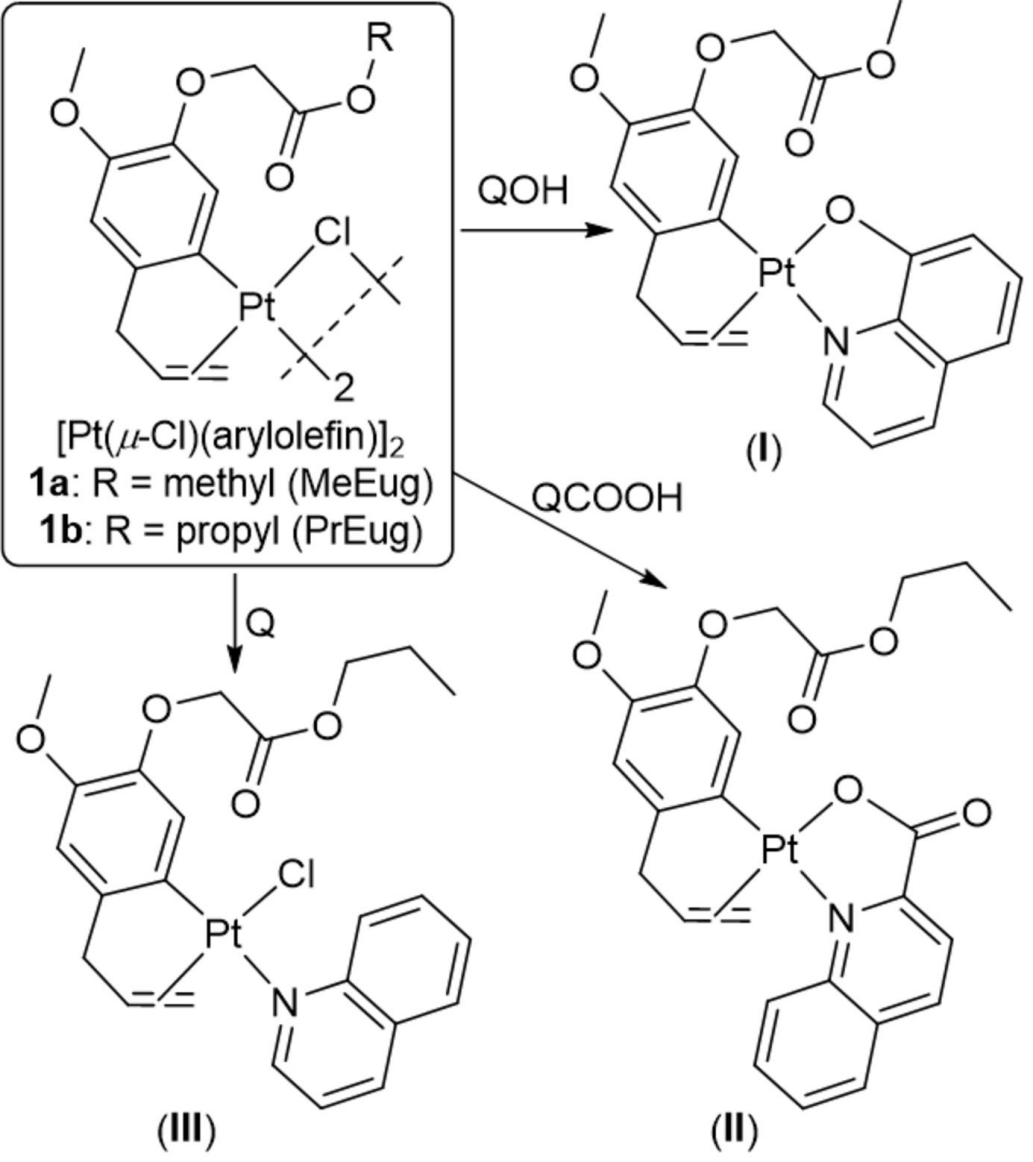
Preparation of organoplatinum(II) complexes (**I**)–(**III**).

In this paper, the synthesis and crystal structure of three organoplatinum(II) complexes containing a natural aryl­olefin, namely (*η* ^2^-2-allyl-4-meth­oxy-5-{[(meth­yloxy)carbon­yl]meth­oxy}phenyl-*Cκ* ^1^)(quinolin-8-olato-*κ* ^2^ *N*,*O*)platinum(II), [Pt(C_13_H_15_O_4_)(C_9_H_6_NO)], (**I**), (*η* ^2^-2-allyl-4-meth­oxy-5-{[(propan-1-yl­oxy)carbon­yl]meth­oxy}phenyl-*Cκ* ^1^)(quinolin-2-carboxyl­ato-*κ* ^2^ *N*,*O*)platinum(II), [Pt(C_15_H_19_O_4_)(C_10_H_6_NO_2_)], (**II**) and (*η* ^2^-2-allyl-4-meth­oxy-5-{[(propan-1-yl­oxy)carbon­yl]meth­oxy}phenyl-*Cκ* ^1^)chlorido­(quinolin-*κ* ^1^ *N*)platinum(II), [Pt(C_15_H_19_O_4_)Cl(C_9_H_7_N)], (**III**), are reported. Complexes (**I**)–(**III**) were synthesized by the reaction between the dimer complexes (**1a**/**1b**) and amine (QOH/QCOOH/Q with Q = quinoline) in an ethanol/acetone solvent with the molar ratio of the dimer complex:amine being 1:2 (Fig. 1 ▸). The crystals of complexes (**I**)–(**III**) were obtained in high yields of 82–87% and were suitable for X-ray diffraction studies. 
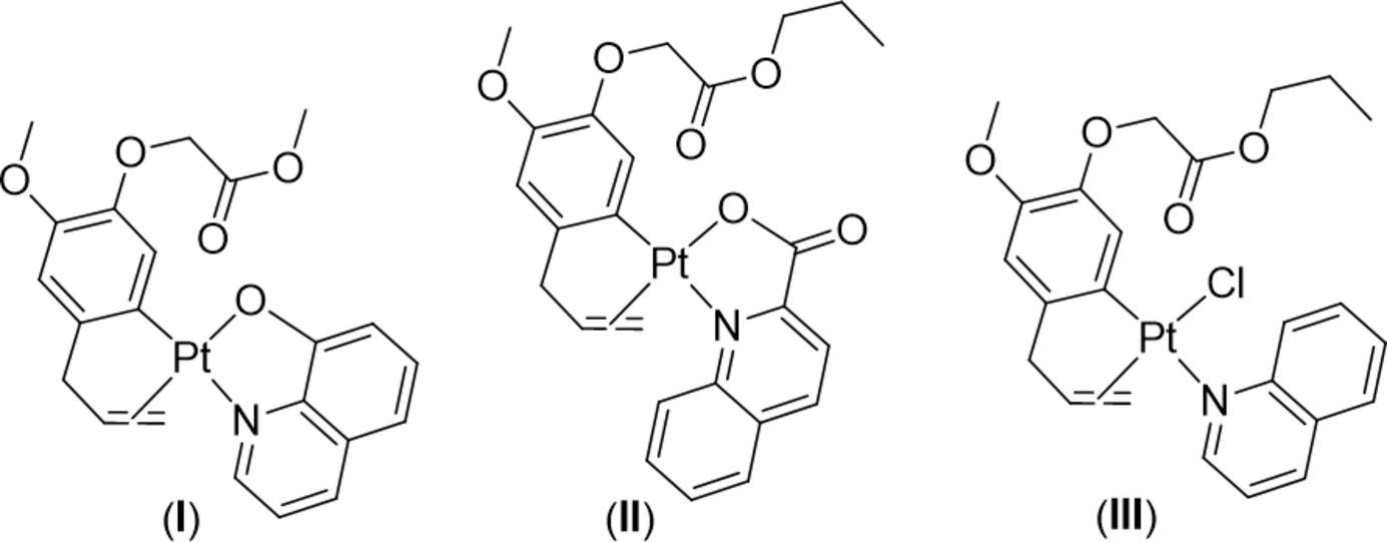


The assigned results of the IR and ^1^H NMR spectra (see section 5) show that the amines cleave the dimers to form monomeric complexes (**I**)–(**III**), in which the amines coordinate with Pt^II^ through the N atoms. For QOH and QCOOH, they were deprotonated at the OH/COOH group and further bonded with Pt^II^ *via* the O atom to produce the chelating complexes (**I**) and (**II**). These conclusions were further strengthened by the single-crystal XRD results. Moreover, the XRD results indicate that the donor N atoms of the amine ligands and the allyl group of aryl­olefin in complexes (**I**)–(**III**) are in the *cis* position with respect to each other.

## 2. Structural commentary

**Figure 2:**
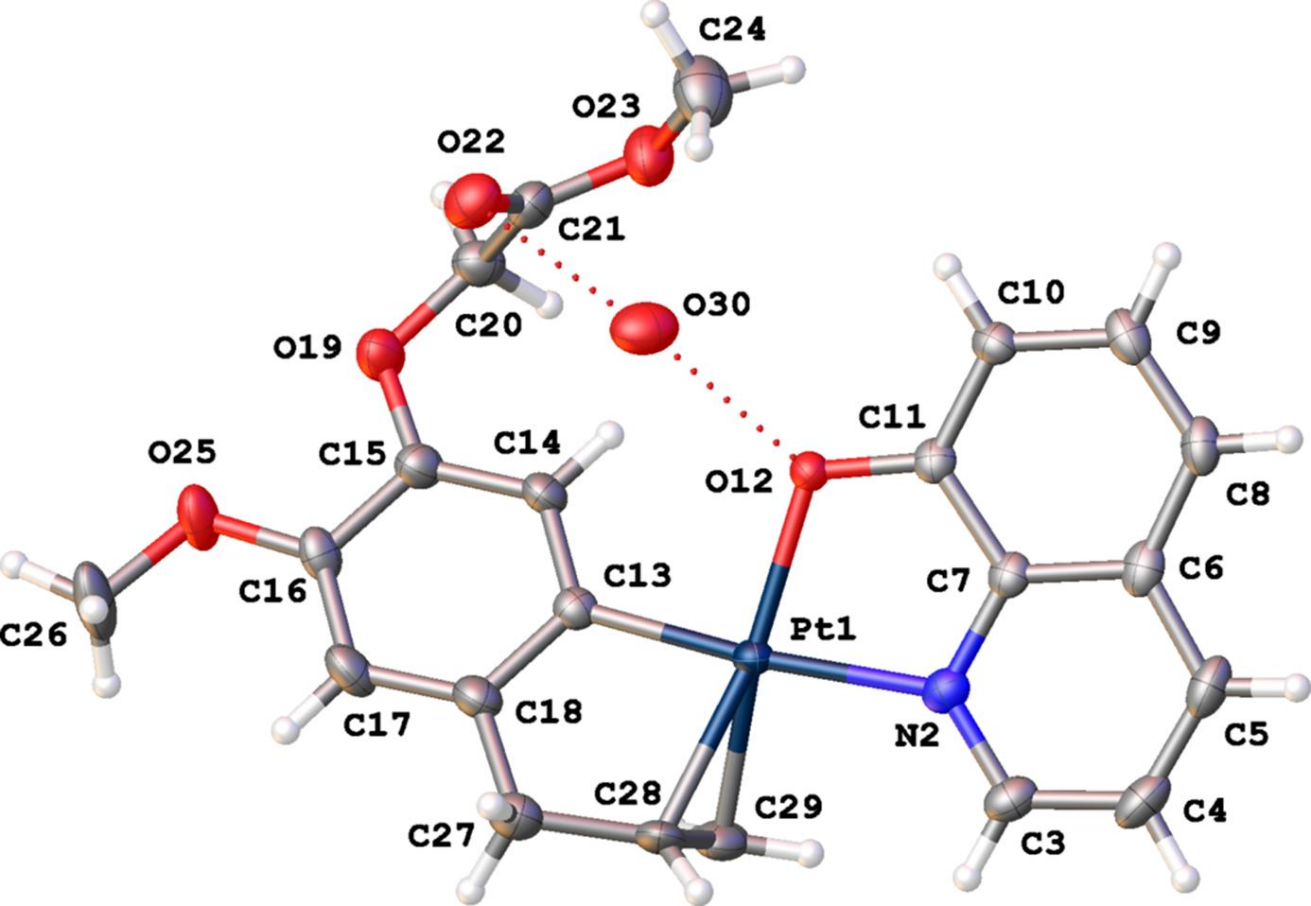
The mol­ecular structure of (**I**), showing the atom-labeling scheme and displacement ellipsoids at the 30% probability level. Water oxygen atom O30 [occupancy 0.473 (11)] is in close contact with atoms O12 and O22 (red dotted lines).

Complex (**I**) crystallizes in the monoclinic space group *P*2_1_/*c* with one complex and a water mol­ecule with partial occupancy of 0.473 (11) in the asymmetric unit (Fig. 2 ▸). No hydrogen atoms could be located for this water mol­ecule, the oxygen atom O30 is in close contact with O12 [O30⋯O12 = 2.718 (8) Å] and O22 [O30⋯O22 = 2.945 (8) Å] suggesting the likelihood that the water forms hydrogen bonds to O12 and O22. The central Pt^II^ atom displays a distorted square-planar coordination with the N2 and O12 atoms of the quinolin-8-olate ligand and the C13 atom and C=C double bond of the aryl­olefin as coordination sphere. The Pt^II^ atom deviates by 0.012 (1) Å from the best plane through atoms N2, O12, C13 and the midpoint of the double bond (r.m.s. deviation = 0.005 Å). The C=C double bond and N2 atom are *cis* with respect to each other. The aryl­olefin ring C13–C18 (r.m.s. deviation = 0.007 Å) makes a dihedral angle of 25.79 (11)° with the best plane through the quinoline ring system (r.m.s. deviation = 0.014 Å).

**Figure 3:**
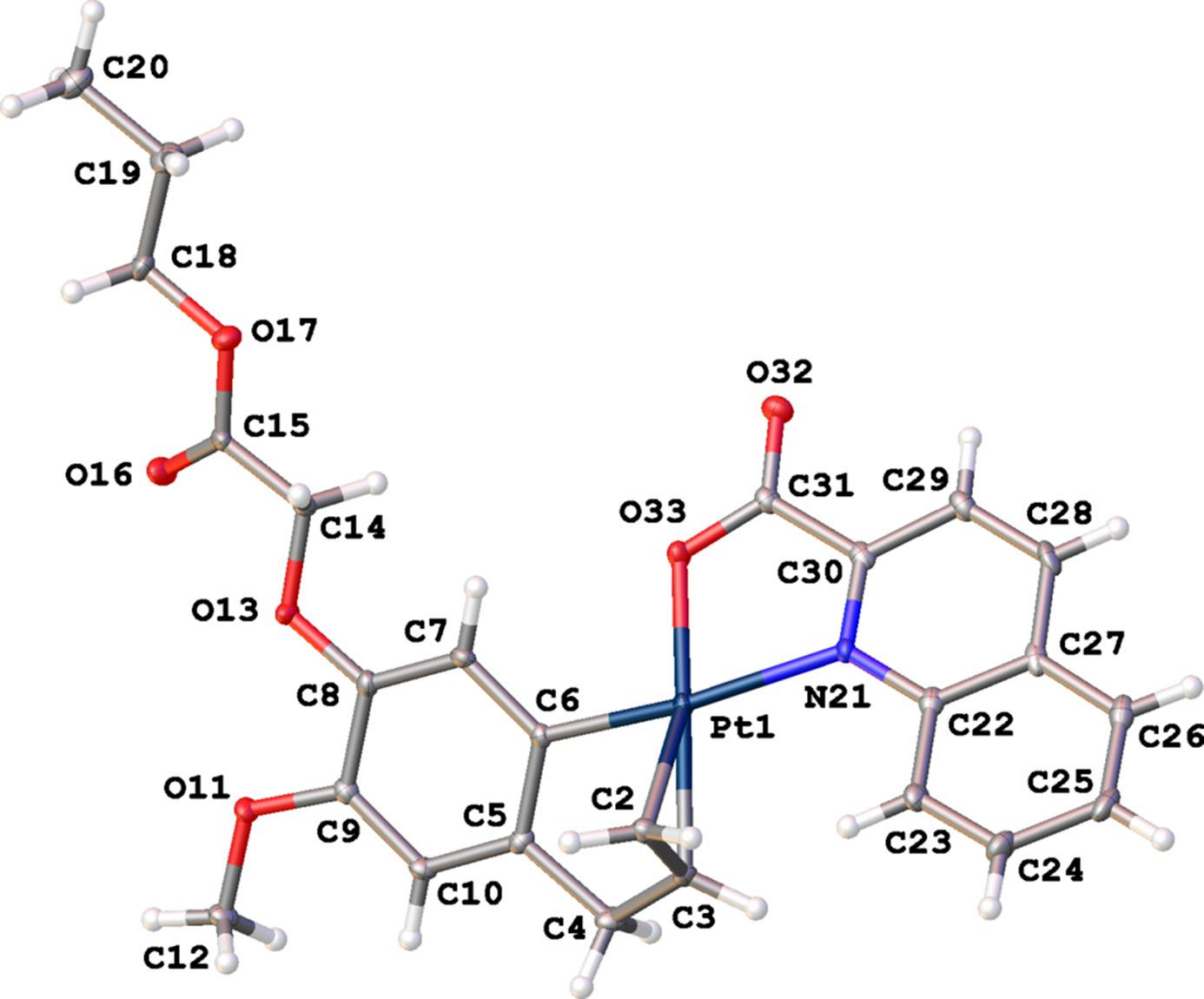
The mol­ecular structure of (**II**), showing the atom-labeling scheme and displacement ellipsoids at the 30% probability level.

Crystals of complex (**II**) crystallize in the monoclinic space group *P*2_1_/*n* with one mol­ecule in the asymmetric unit (Fig. 3 ▸). The *cis* position of quinoline N atom and the allyl group and the coordination of the Pt^II^ atom is similar to that in (**I**) with a deviation of Pt^II^ of 0.033 (1) Å from the best plane through atoms N21, O33, C6 and the midpoint of the double bond. The dihedral angle between the best planes through the C5–C10 ring (r.m.s. deviation = 0.008 Å) and through the quinoline ring system (r.m.s. deviation = 0.048 Å) is 41.72 (16)°.

**Figure 4:**
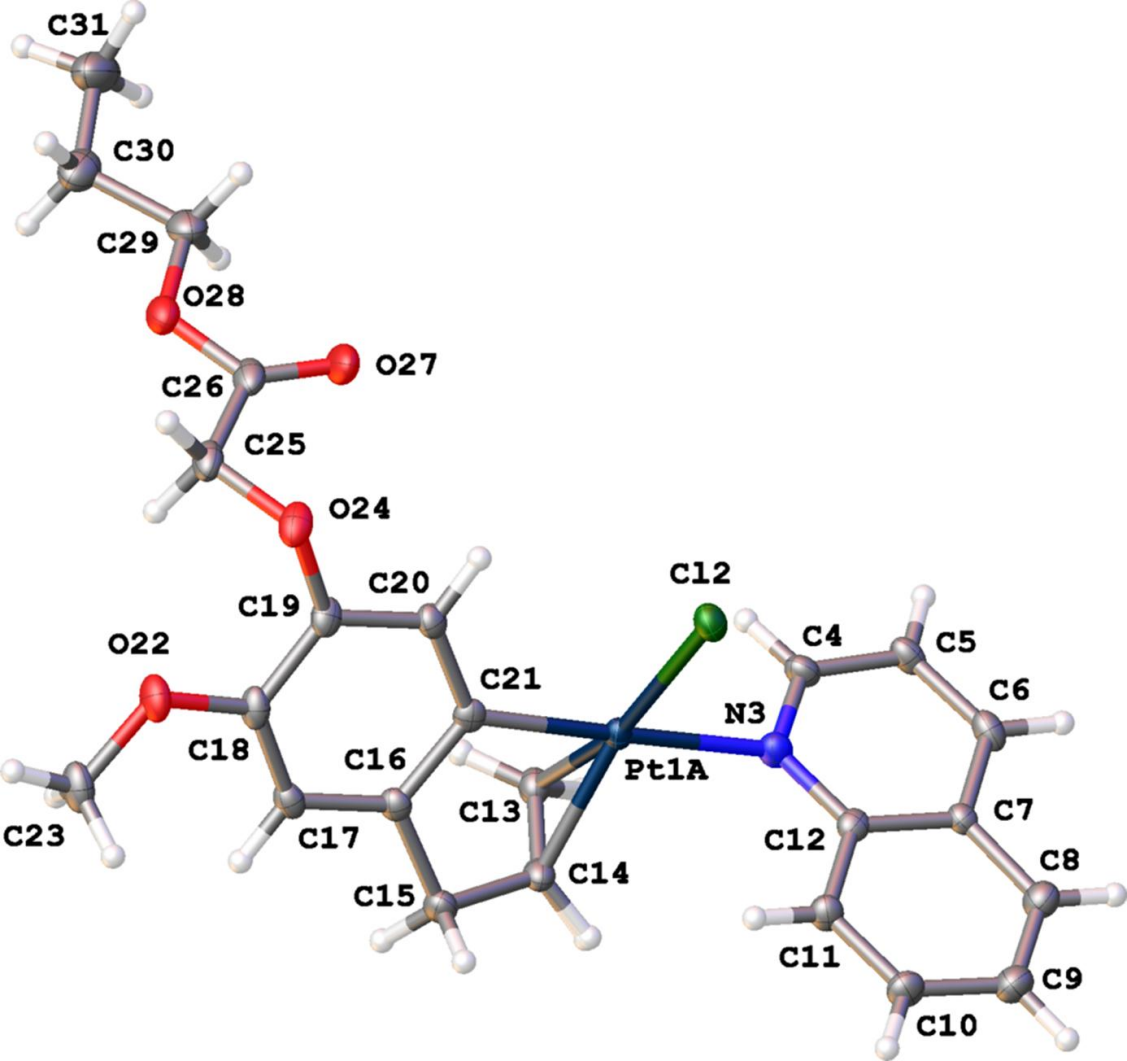
The mol­ecular structure of (**III**), showing the atom-labeling scheme and displacement ellipsoids at the 30% probability level. Only the major position of the disordered Pt atom is shown.

Complex (**III**) crystallizes in the monoclinic space group *P*2_1_/*c* with one complex in the asymmetric unit (Fig. 4 ▸). The Pt^II^ atom was found to be disordered over two positions with refined occupancies of 0.928 (7) and 0.072 (7) and a distance between both Pt components of 0.529 (17) Å. In the subsequent discussion, only the main position of the disordered Pt atom is used. The distorted square-planar coordination of the Pt^II^ atom is again characterized by a *cis* position of the C=C double bond and atom N3. The Pt^II^ atom deviates by 0.005 (1) Å from the best plane through atoms Cl2, N3, C21 and the midpoint of the double bond (r.m.s. deviation = 0.026 Å). Complex (**III**) displays a short intra­molecular contact O22⋯H25*B* (2.40 Å) resulting from a different orientation of the side chain at C19 compared to complexes (**I**) and (**II**). This is further illustrated by the different torsion angles determining the orientation of the side chain in the three complexes: 178.4 (4)° for C16—C15—O19—C20 in (**I**), 179.8 (4)° for C9—C8—O13—C14 (**II**), and −69.9 (5)° for C18—C19—O24—C25 (**III**). Compared to the two other complexes, the C16–C21 aryl­olefin ring (r.m.s. deviation = 0.013 Å) makes a larger dihedral angle of 57.38 (18)° with the best plane through the quinoline ring system (r.m.s. deviation = 0.017 Å).

## 3. Supra­molecular features

**Figure 5:**
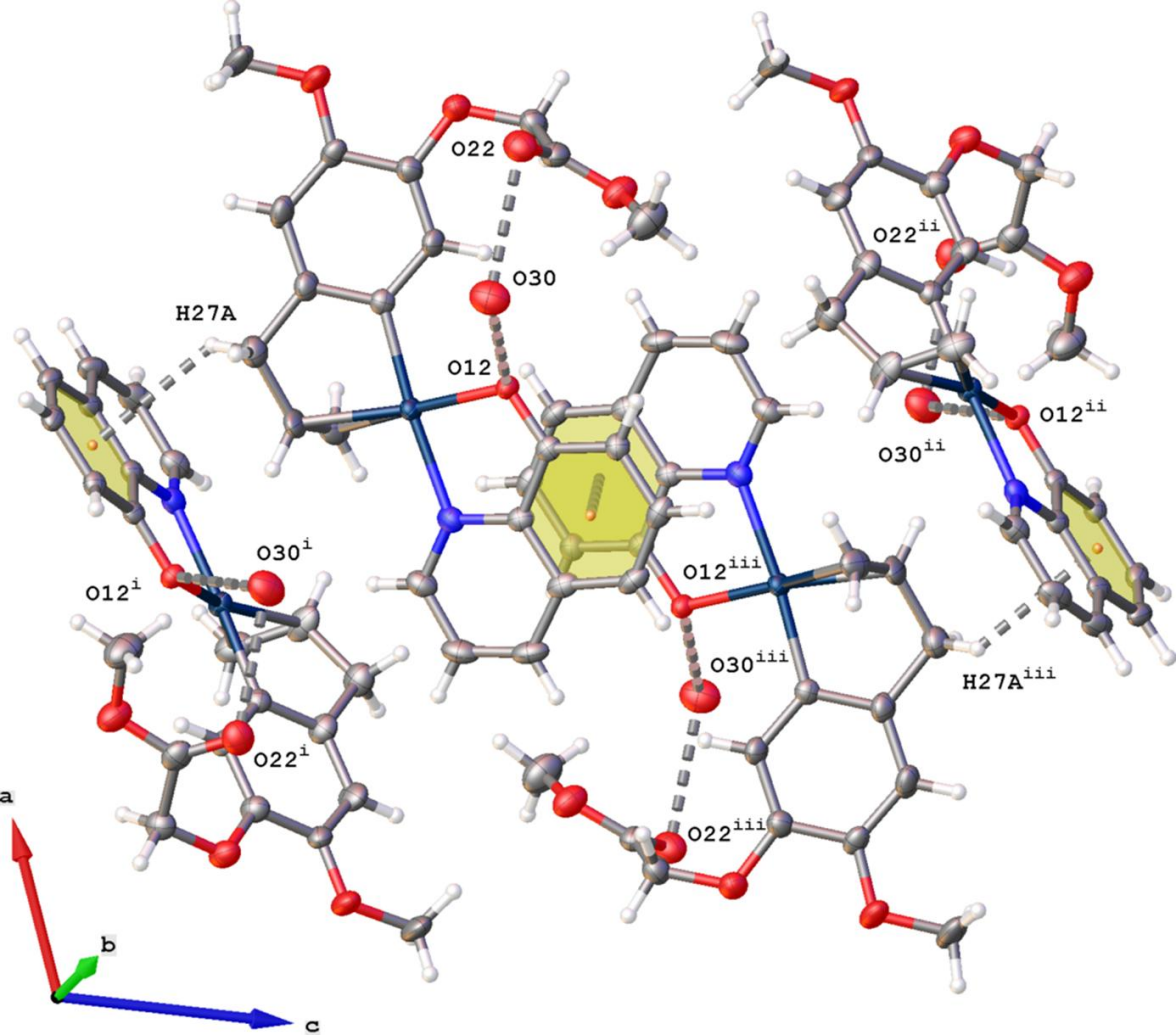
Partial packing diagram for (**I**) showing the π–π and C—H⋯π inter­actions (gray dashed lines). The centroids of the C6–C11 rings are shown as orange dots. [Symmetry codes: (i) −*x*, − 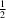 + *y*, 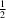 − *z*; (ii) *x*, 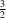 − *y*, 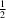 + *z*; (iii) −*x*, 1 − *y*, 1 − *z*.]

The crystal packing of (**I**) is characterized by π–π and C—H⋯π inter­actions (Fig. 5 ▸). The shortest centroid–centroid distance is observed for the stacking of rings C6–C11 resulting in inversion dimers [*Cg*⋯*Cg* ^i^ = 3.566 (2) Å; slippage = 1.369 Å; symmetry code: (i) −*x*, 1 − *y*, 1 − *z*]. Neighboring dimers are connected in the *c*-axis direction *via* C—H⋯π inter­actions of the same ring with C27—H27*A* (Table 1 ▸). As mentioned above, oxygen atom O30 [occupancy 0.473 (11)] occupies a small cavity in the packing and is in close contact with atoms O12 and O22.

**Figure 6:**
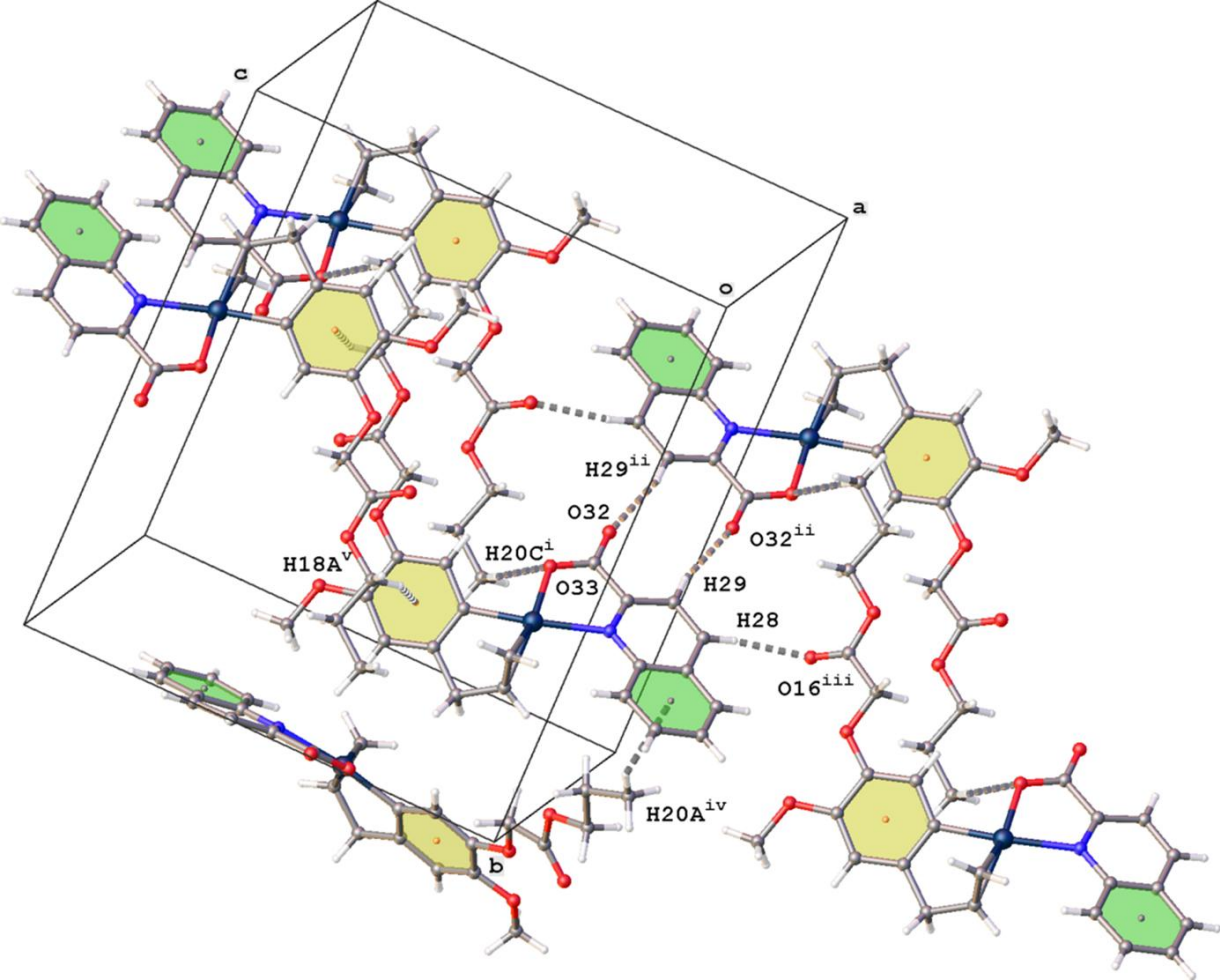
Partial packing diagram for (**II**) showing the C—H⋯O and C—H⋯π inter­actions (gray dashed lines). The centroids of rings C5–C10 (*Cg*1) and C22–C27 (*Cg*2) are shown as orange and gray dots, respectively. [Symmetry codes: (i) 1 − *x*, 1 − *y*, 1 − *z*; (ii) 1 − *x*, 1 − *y*, −*z*; (iii) *x*, *y*, −1 + *z*; (iv) 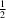 − *x*, 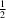 + *y*, 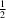 − *z*; (v) −*x*, 1 − *y*, 1 − *z*.]

In the crystal, mol­ecules of (**II**) are connected by C—H⋯O and C—H⋯π inter­actions (Fig. 6 ▸). Inversion dimers are formed by C29—H29⋯O32 inter­actions. These dimers are further linked by C20—H20*C*⋯O33, C28—H28⋯O16, C18—H18*A*⋯π and C20—H20*A*⋯π inter­actions. Details are given in Table 2 ▸. No π–π inter­actions are present in the packing, but a short contact distance between Pt1 and ring N21,C22,C27–C30 is noted [*Cg*3⋯Pt1^vi^ = 3.670 (2) Å; *Cg*3 is the centroid of ring N21,C22,C27–C30; symmetry code: (vi) −*x*, 1 − *y*, −*z*].

**Figure 7:**
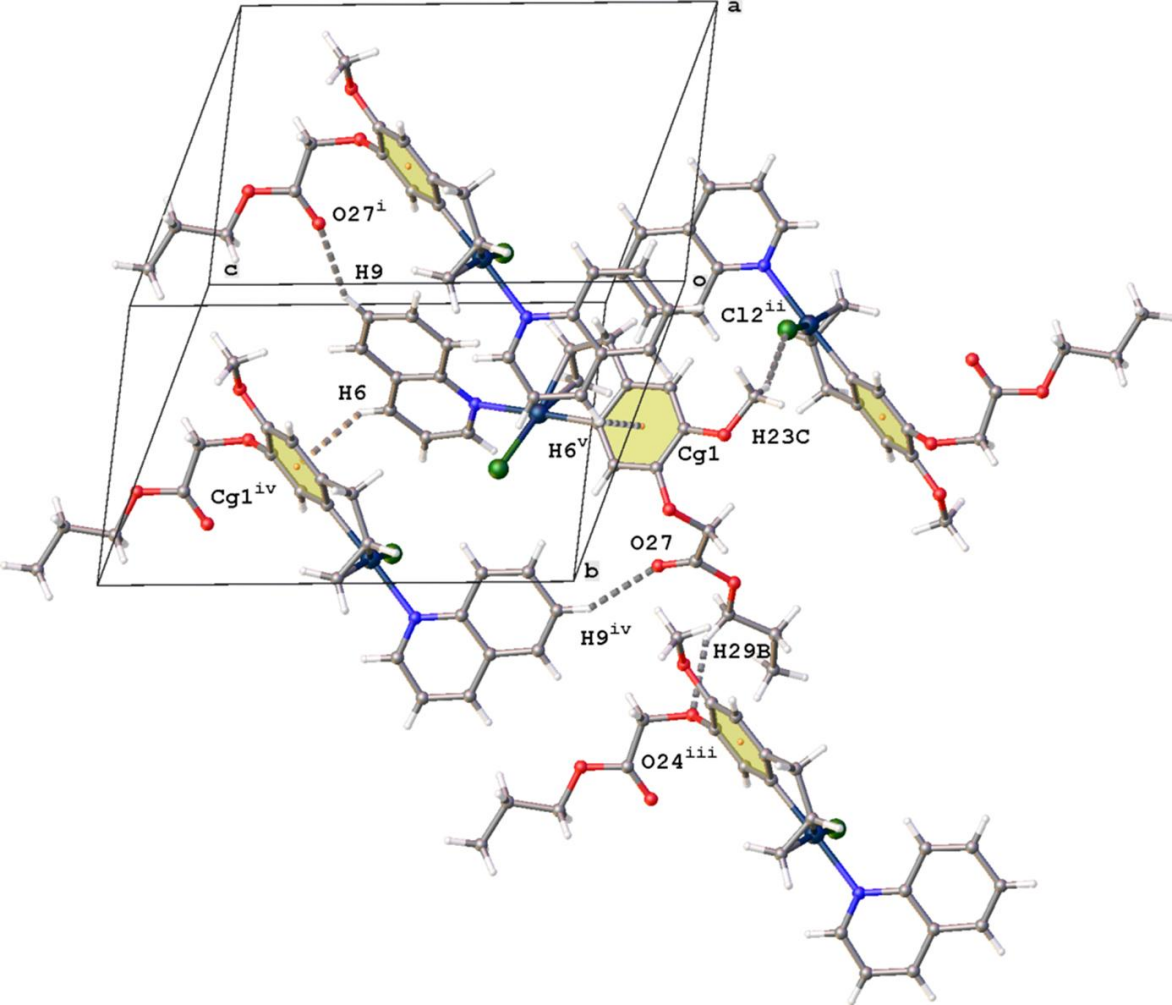
Partial packing diagram for (**III**) showing the C—H⋯O, C—H⋯Cl and C—H⋯π inter­actions (gray dashed lines). Only the major position of the disordered Pt atom is shown. [Symmetry codes: (i) 1 − *x*, − 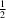 + *y*, − 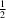 − *z*; (ii) *x*, 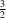 − *y*, − 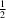 + *z*; (iii) −*x*, 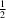 + *y*, − 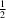 − *z*; (iv) 1 − *x*, 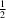 + *y*, 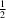 − *z*; (v) 1 − *x*, − 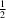 + *y*, 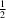 − *z*.]

For complex (**III**), the mol­ecules are linked together by C—H⋯O, C—H⋯Cl and C—H⋯π inter­actions (Fig. 7 ▸, Table 3 ▸). Atoms H6 and H9 of the quinoline ring system inter­act with ring C16–C21 and O27, respectively. At the other end of the complex, the meth­oxy group links with a neighboring Cl2 atom and the prop­yloxy group connects with an neighboring atom O24. Again, despite the presence of aromatic rings, no π–π inter­actions are observed in the packing.

## 4. Database survey

A search of the Cambridge Structural Database (CSD, Version 5.45, update of March 2024; Groom *et al.*, 2016 ▸) for Pt complexes coordinated to C=C, C, N and O or Cl resulted in 15 hits. For three hits, the N-containing ligand is a quinoline derivative: {5-(2-eth­oxy-2-oxoeth­oxy)-4-meth­oxy-2-[prop-2-en-1-yl]phen­yl}(2-methyl­quinolin-8-olato)platinum(II) (ref­code LOJDEW; Hai *et al.*, 2019 ▸), [η^2^-4,5-dimeth­oxy-2-(prop-2-en-1-yl)phen­yl](quinolin-8-olato)platinum(II) (refcode GACYUH; Bui *et al.*, 2016 ▸) and [5-(2-eth­oxy-2-oxoeth­oxy)-4-meth­oxy-2-(prop-2-en-1-yl)phen­yl](quinoline-2-carboxyl­ato)platinum(II) (refcode MEKGER; Da *et al.*, 2015 ▸).

**Figure 8:**
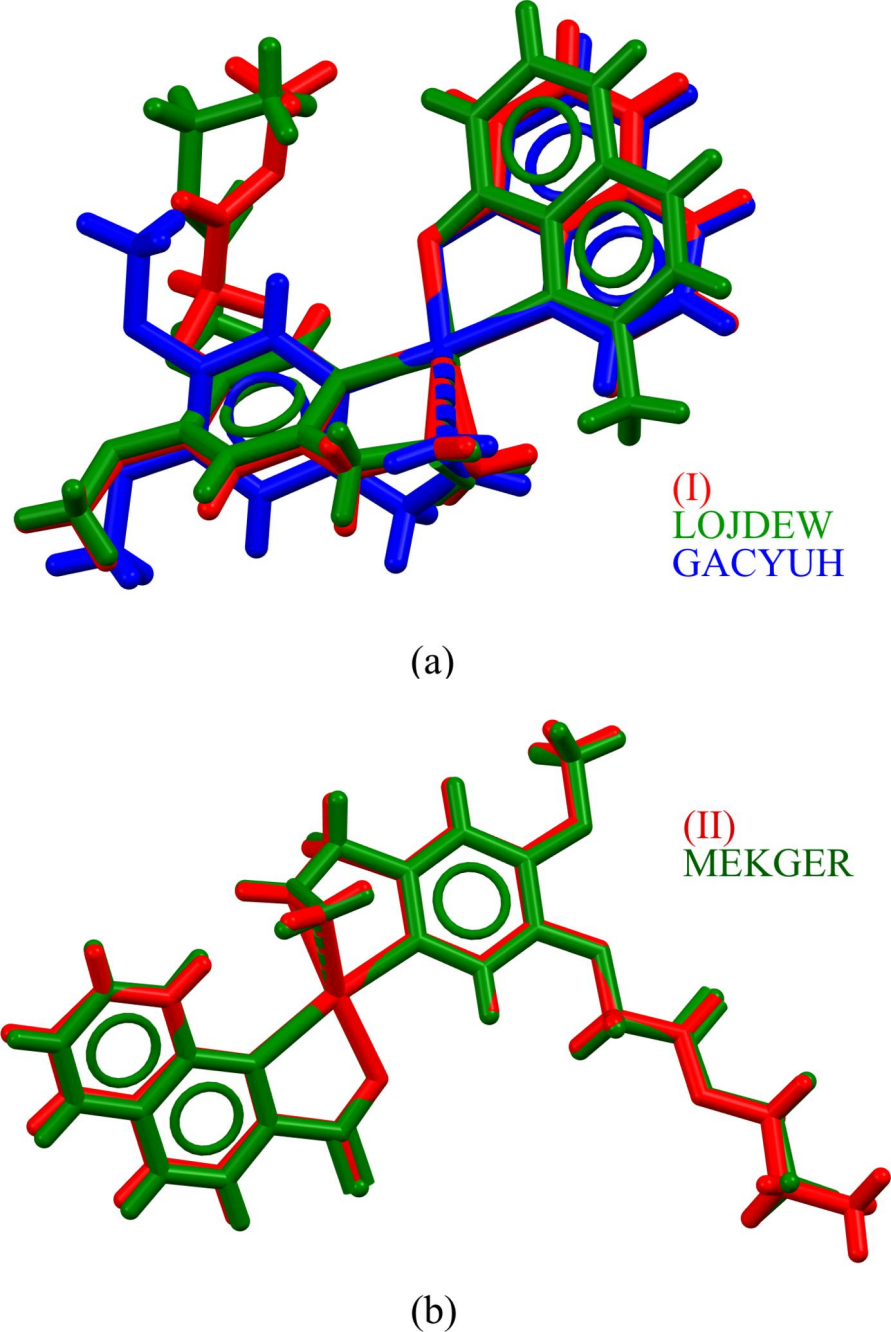
Overlay of the Pt, N, O, C and C=C atoms in (*a*) (**I**) (red), LOJDEW (green) and GACYUH (blue), and (*b*) (**II**) (red) and MEKGER (green).

Entries LOJDEW and GACYUH are comparable to complex (**I**), but crystallize with different unit cells. An overlay of Pt and its coordination sphere (N, O, C, C=C) gives for (**I**) and LOJDEW an r.m.s. deviation of 0.106 Å, and for (**I**) and GACYUH 0.120 Å (Fig. 8 ▸ *a*). Compared to (**II**) and LOJDEW, the double bond of the allyl chain in GACYUH complexes is in a different orientation with Pt. This causes also a different orientation of the aromatic ring of the aryl­olefin ligand.

Entry MEKGER is comparable to complex (**II**) and both structures are isomorphous. The somewhat longer *b* axis in (**II**) (18.500 *versus* 17.326 Å) is caused by the longer propyl chain (compared to ethyl in MEKGER), which is oriented in the *b*-axis direction. The r.m.s. deviation for an overlay of Pt and its coordination sphere is 0.0371 Å (Fig. 8 ▸ *b*).

## 5. Synthesis and crystallization

The synthetic protocol for complexes (**I**)–(**III**) is shown in Fig. 1 ▸. The starting complexes [Pt(*μ*-Cl)(MeEug)]_2_ and [Pt(*μ*-Cl)(PrEug)]_2_ were synthesized according to the procedures of Da *et al.* (2010 ▸) and Chi *et al.* (2013 ▸).

**Synthesis of complex [Pt(MeEug)(QO)] (I).** A solution of 8-hy­droxy­quinoline (15 mg, 0.1 mmol) in 3 mL of ethanol was dropped into a suspension of [Pt(*μ*-Cl)(MeEug)]_2_ (47 mg, 0.05 mmol) in 2 mL of acetone. The reaction mixture was stirred at ambient temperature (AT) for 2 h until a clear solution was obtained. Orange crystals suitable for X-ray diffraction were obtained by slow evaporation of the solvent of the obtained solution at AT within 12 h. The yield was 47 mg (82%). ^1^H NMR (chloro­form-*d*_1_, 500 MHz): δ 8.33 (*d*, ^3^ *J* = 8.0 Hz, 1H, Ar-H), 8.11 (*d*, ^3^ *J* = 4.5 Hz, 1H, Ar-H), 8.56 (*t*, ^3^ *J* = 8.0 Hz, 1H, Ar-H), 7.46 (*dd*, ^3^ *J* = 8.0 Hz, 4.5 Hz, 1H, Ar-H), 7.26 (*d*, ^3^ *J* = 8.0 Hz, 1H, Ar-H), 7.08 (*d*, ^3^ *J* = 8.0 Hz, 1H, Ar-H), 7.10 (*s*, 1H, Ar-H), 6.69 (*s*, 1H, Ar-H), 4.78 (*s*, 2H, OCH_2_), 4.74 (*m*, 1H, C*H*=CH_2_), 4.06 (*d*, ^3^ *J* = 7.5 Hz, ^2^ *J*_PtH_ = 60 Hz, 1H, CH=C*H*_2_), 3.85 (*s*, 3H, CH_3_), 3.83 (*ov*, 4H, CH=C*H*_2_, OCH_3_), 3.72 (*dd*, ^2^ *J* = 16.5 Hz, ^3^ *J* = 6.0 Hz, 1H, CH_2_), 2.86 (*d*, ^2^ *J* = 16.5 Hz, 1H, CH_2_). FT–IR (KBr pellet, cm^−1^): 2928 (CH), 1751 (C=O), 1578, 1497 (C=C).

**Synthesis of complex [Pt(PrEug)(QCOO)] (II).** This complex was prepared starting from [Pt(*μ*-Cl)(PrEug)]_2_ (49 mg, 0.05 mmol) and quinoline-2-carb­oxy­lic acid (18 mg, 0.1 mmol) according to the procedure for the synthesis of (**I**). The yield was 54 mg (85%), and the orange crystals obtained were suitable for X-ray diffraction. ^1^H NMR (acetone-*d*_6_, 500 MHz): δ 8.86 (*d*, ^3^ *J* = 8.0 Hz, 1H, Ar-H), 8.30 (*d*, ^3^ *J* = 8.0 Hz, 1H, Ar-H), 8.27 (*d*, ^3^ *J* = 8.0 Hz, 1H, Ar-H), 8.09 (*m*, 1H, Ar-H), 7.89 (*t*, ^3^ *J* = 7.0 Hz, 1H, Ar-H), 7.77 (*d*, ^3^ *J* = 8.0 Hz, 1H, Ar-H), 7.02 (*s*, ^3^ *J*_PtH_ = 40 Hz, 1H, Ar-H), 6.77 (*s*, 1H, Ar-H), 5.75 (*m*, ^2^ *J*_PtH_ = 70 Hz, 1H, C*H*=CH_2_), 4.71 (*d*, ^3^ *J* = 7.5 Hz, ^2^ *J*_PtH_ = 60 Hz, 1H, CH=C*H*_2_), 4.67 (*s*, 2H, OCH_2_), 4.19 (*m*, 2H, C*H*_2_CH_2_CH_3_), 3.94 (*d*, ^3^ *J* = 13.5 Hz, ^2^ *J*_PtH_ = 65 Hz, 1H, CH=C*H*_2_), 3.82–3.78 (*ov*, 4H, CH_2_, OCH_3_), 1.74 (*m*, 2H, CH_2_C*H*_2_CH_3_), 0.97 (*t*, ^3^ *J* = 7.0 Hz, 3H, CH_2_CH_2_C*H*_3_). FT–IR (KBr pellet, cm^−1^): 3030, 2925 (CH), 1750, 1666 (C=O), 1593, 1465 (C=C).

**Synthesis of complex [PtCl(PrEug)(Q)] (III).** This complex was prepared starting from [Pt(*μ*-Cl)(PrEug)]_2_ (49 mg, 0.05 mmol) and quinoline (12 µL, 0.1 mmol) according to the procedure for the synthesis of (**I**). The yield was 54 mg (87%), and the yellow crystals obtained were suitable for X-ray diffraction. ^1^H NMR (acetone-*d*_6_, 500 MHz): δ 9.06 (*ov*, 2H, Ar-H), 8.52 (*d*, ^3^ *J* = 8.0 Hz, 1H, Ar-H), 8.04 (*d*, ^3^ *J* = 8.0 Hz, 1H, Ar-H), 7.89 (*m*, 1H, Ar-H), 7.67–7.61 (*ov*, 2H, Ar-H), 7.0 (*s*, ^3^ *J*_PtH_ = 40 Hz, 1H, Ar-H), 6.58 (*s*, 1H, Ar-H), 4.65 (*br*, 1H, C*H*=CH_2_), 4.49 (*s*, 2H, OCH_2_), 4.0 (*t*, ^3^ *J* = 7.0 Hz, 2H, C*H*_2_CH_2_CH_3_), 3.74–3.62 (*ov*, 6H, CH=C*H*_2_, CH_2_, OCH_3_), 2.55 (*d*, ^2^ *J* = 16.5 Hz, 1H, CH_2_), 1.56 (*m*, 2H, CH_2_C*H*_2_CH_3_), 0.81 (*t*, ^3^ *J* = 7.0 Hz, 3H, CH_2_CH_2_C*H*_3_). FT–IR (KBr pellet, cm^−1^): 3060, 2936 (CH), 1745 (C=O), 1576, 1471 (C=C).

## 6. Refinement

Crystal data, data collection and structure refinement details are summarized in Table 4 ▸. Non-hydrogen atoms were refined anisotropically. Hydrogen atoms were included as riding contributions in idealized positions with isotropic displacement parameters *U*_iso_(H) = 1.2 *U*_eq_(C) (1.5 for methyl groups). The Pt atom in (**III**) was found to be disordered over two positions with refined occupancies of 0.928 (7) and 0.072 (7).

## Supplementary Material

Crystal structure: contains datablock(s) I, II, III. DOI: 10.1107/S2056989024004572/pk2707sup1.cif

Structure factors: contains datablock(s) I. DOI: 10.1107/S2056989024004572/pk2707Isup2.hkl

Structure factors: contains datablock(s) II. DOI: 10.1107/S2056989024004572/pk2707IIsup3.hkl

Structure factors: contains datablock(s) III. DOI: 10.1107/S2056989024004572/pk2707IIIsup4.hkl

CCDC references: 2355693, 2355692, 2355691

Additional supporting information:  crystallographic information; 3D view; checkCIF report

## Figures and Tables

**Table 1:** Hydrogen-bond geometry (Å, °) for (**I**) *Cg*1 is the centroid of ring C6–C11.

*D*—H⋯*A*	*D*—H	H⋯*A*	*D*⋯*A*	*D*—H⋯*A*
C27—H27*A*⋯*Cg*1^i^	0.97	2.81	3.465 (4)	125

Symmetry code: (i) 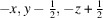 .

**Table 2:** Hydrogen-bond geometry (Å, °) for (**II**) *Cg*1 and *Cg*2 are the centroids of rings C5–C10 and C22–C27, respectively.

*D*—H⋯*A*	*D*—H	H⋯*A*	*D*⋯*A*	*D*—H⋯*A*
C20—H20*C*⋯O33^i^	0.96	2.52	3.462 (6)	168
C28—H28⋯O16^ii^	0.93	2.26	3.159 (5)	164
C29—H29⋯O32^iii^	0.93	2.43	3.334 (6)	166
C18—H18*A*⋯*Cg*1^iv^	0.97	2.97	3.711 (5)	134
C20—H20*A*⋯*Cg*2^v^	0.96	2.78	3.605 (6)	144

Symmetry codes: (i) 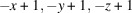 ; (ii) 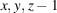 ; (iii) 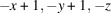 ; (iv) 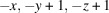 ; (v) 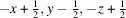 .

**Table 3:** Hydrogen-bond geometry (Å, °) for (**III**) *Cg*1 is the centroid of ring C16–C21.

*D*—H⋯*A*	*D*—H	H⋯*A*	*D*⋯*A*	*D*—H⋯*A*
C9—H9⋯O27^i^	0.95	2.59	3.445 (5)	150
C23—H23*C*⋯Cl2^ii^	0.98	2.70	3.618 (6)	157
C29—H29*B*⋯O24^iii^	0.99	2.50	3.381 (7)	148
C6—H6⋯*Cg*1^iv^	0.95	2.73	3.269 (5)	117

Symmetry codes: (i) 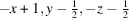 ; (ii) 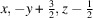 ; (iii) 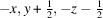 ; (iv) 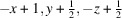 .

**Table 4:** Experimental details

	(**I**)	(**II**)	(**III**)
Crystal data			
Chemical formula	[Pt(C_13_H_15_O_4_)(C_9_H_6_NO)]	[Pt(C_15_H_19_O_4_)(C_10_H_6_NO_2_)]	[Pt(C_15_H_19_O_4_)Cl(C_9_H_7_N)]
*M*_r_	582.49	630.55	623.00
Crystal system, space group	Monoclinic, *P*2_1_/*c*	Monoclinic, *P*2_1_/*n*	Monoclinic, *P*2_1_/*c*
Temperature (K)	100	100	114
*a*, *b*, *c* (Å)	13.1510 (4), 8.5584 (2), 18.2071 (6)	8.2857 (4), 18.5001 (9), 14.6282 (7)	14.576 (2), 11.0945 (9), 15.700 (2)
β (°)	105.714 (3)	102.014 (5)	117.197 (18)
*V* (Å^3^)	1972.65 (10)	2193.18 (19)	2258.1 (6)
*Z*	4	4	4
Radiation type	Mo *K*α	Mo *K*α	Mo *K*α
μ (mm^−1^)	7.15	6.44	6.36
Crystal size (mm)	0.3 × 0.15 × 0.1	0.4 × 0.4 × 0.3	0.27 × 0.2 × 0.16

Data collection			
Diffractometer	SuperNova, Single source at offset, Eos	SuperNova, Single source at offset, Eos	SuperNova, Single source at offset, Eos
Absorption correction	Multi-scan (*CrysAlis PRO*; Rigaku OD, 2022 ▸)	Multi-scan (*CrysAlis PRO*; Rigaku OD, 2022 ▸)	Multi-scan (*CrysAlis PRO*; Rigaku OD, 2022 ▸)
*T*_min_, *T*_max_	0.300, 1.000	0.669, 1.000	0.579, 1.000
No. of measured, independent and observed [*I* > 2σ(*I*)] reflections	41833, 4046, 3672	23965, 5366, 4652	8975, 4603, 3885
*R*_int_	0.039	0.057	0.028
(sin θ/λ)_max_ (Å^−1^)	0.625	0.685	0.625

Refinement			
*R*[*F* ^2^ > 2σ(*F* ^2^)], *wR*(*F* ^2^), *S*	0.021, 0.054, 1.10	0.033, 0.070, 1.05	0.030, 0.056, 1.04
No. of reflections	4046	5366	4603
No. of parameters	274	300	292
No. of restraints	0	0	288
H-atom treatment	H-atom parameters constrained	H-atom parameters constrained	H-atom parameters constrained
Δρ_max_, Δρ_min_ (e Å^−3^)	0.90, −0.60	2.16, −1.73	0.85, −1.07

Computer programs: *CrysAlis PRO* (Rigaku OD, 2022 ▸), *SHELXS* (Sheldrick, 2008 ▸), *SHELXL* (Sheldrick, 2015 ▸) and *OLEX2* (Dolomanov *et al.*, 2009 ▸).

## supplementary crystallographic information

### [4-Methoxy-5-(2-methoxy-2-oxoethoxy)-2-(prop-2-en-1-yl)phenyl](quinolin-8-olato)platinum(II) (I) Crystal data

**Table:**

[Pt(C_13_H_15_O_4_)(C_9_H_6_NO)]	*F*(000) = 1127.2
*M**_r_* = 582.49	*D*_x_ = 1.961 Mg m^−^^3^
Monoclinic, *P*2_1_/*c*	Mo *K*α radiation, λ = 0.71073 Å
*a* = 13.1510 (4) Å	Cell parameters from 19506 reflections
*b* = 8.5584 (2) Å	θ = 2.9–29.0°
*c* = 18.2071 (6) Å	µ = 7.15 mm^−^^1^
β = 105.714 (3)°	*T* = 100 K
*V* = 1972.65 (10) Å^3^	Plate, light brown
*Z* = 4	0.3 × 0.15 × 0.1 mm

### [4-Methoxy-5-(2-methoxy-2-oxoethoxy)-2-(prop-2-en-1-yl)phenyl](quinolin-8-olato)platinum(II) (I) Data collection

**Table:**

SuperNova, Single source at offset, Eos diffractometer	4046 independent reflections
Radiation source: micro-focus sealed X-ray tube, SuperNova (Mo) X-ray Source	3672 reflections with *I* > 2σ(*I*)
Mirror monochromator	*R*_int_ = 0.039
Detector resolution: 15.9631 pixels mm^-1^	θ_max_ = 26.4°, θ_min_ = 2.4°
ω scans	*h* = −16→16
Absorption correction: multi-scan (CrysAlisPro; Rigaku OD, 2022)	*k* = −10→10
*T*_min_ = 0.300, *T*_max_ = 1.000	*l* = −22→22
41833 measured reflections	

### [4-Methoxy-5-(2-methoxy-2-oxoethoxy)-2-(prop-2-en-1-yl)phenyl](quinolin-8-olato)platinum(II) (I) Refinement

**Table:**

Refinement on *F*^2^	0 restraints
Least-squares matrix: full	Hydrogen site location: inferred from neighbouring sites
*R*[*F*^2^ > 2σ(*F*^2^)] = 0.021	H-atom parameters constrained
*wR*(*F*^2^) = 0.054	*w* = 1/[σ^2^(*F*_o_^2^) + (0.0191*P*)^2^ + 6.3477*P*] where *P* = (*F*_o_^2^ + 2*F*_c_^2^)/3
*S* = 1.10	(Δ/σ)_max_ < 0.001
4046 reflections	Δρ_max_ = 0.90 e Å^−^^3^
274 parameters	Δρ_min_ = −0.60 e Å^−^^3^

### [4-Methoxy-5-(2-methoxy-2-oxoethoxy)-2-(prop-2-en-1-yl)phenyl](quinolin-8-olato)platinum(II) (I) Special details

**Table:**

Geometry. All esds (except the esd in the dihedral angle between two l.s. planes) are estimated using the full covariance matrix. The cell esds are taken into account individually in the estimation of esds in distances, angles and torsion angles; correlations between esds in cell parameters are only used when they are defined by crystal symmetry. An approximate (isotropic) treatment of cell esds is used for estimating esds involving l.s. planes.

### [4-Methoxy-5-(2-methoxy-2-oxoethoxy)-2-(prop-2-en-1-yl)phenyl](quinolin-8-olato)platinum(II) (I) Fractional atomic coordinates and isotropic or equivalent isotropic displacement parameters (Å^2^)

**Table:**

	*x*	*y*	*z*	*U*_iso_*/*U*_eq_	Occ. (<1)
Pt1	0.10321 (2)	0.48502 (2)	0.33132 (2)	0.01955 (6)	
N2	−0.0487 (3)	0.4601 (4)	0.34836 (18)	0.0235 (7)	
C3	−0.1264 (3)	0.3610 (5)	0.3186 (2)	0.0277 (9)	
H3	−0.1190	0.2926	0.2807	0.033*	
C4	−0.2193 (3)	0.3556 (5)	0.3422 (3)	0.0335 (10)	
H4	−0.2720	0.2840	0.3202	0.040*	
C5	−0.2325 (3)	0.4558 (5)	0.3975 (3)	0.0313 (9)	
H5	−0.2937	0.4517	0.4136	0.038*	
C6	−0.1525 (3)	0.5657 (5)	0.4300 (2)	0.0250 (8)	
C7	−0.0605 (3)	0.5634 (4)	0.4035 (2)	0.0206 (7)	
C8	−0.1587 (3)	0.6759 (5)	0.4861 (2)	0.0266 (9)	
H8	−0.2177	0.6783	0.5050	0.032*	
C9	−0.0776 (3)	0.7795 (5)	0.5129 (2)	0.0265 (9)	
H9	−0.0828	0.8525	0.5496	0.032*	
C10	0.0138 (3)	0.7779 (4)	0.4862 (2)	0.0216 (8)	
H10	0.0672	0.8504	0.5049	0.026*	
C11	0.0246 (3)	0.6696 (4)	0.4326 (2)	0.0199 (7)	
O12	0.11072 (19)	0.6615 (3)	0.40694 (14)	0.0202 (5)	
C13	0.2494 (3)	0.5222 (4)	0.3234 (2)	0.0217 (8)	
C14	0.3317 (3)	0.5838 (5)	0.3820 (2)	0.0260 (8)	
H14	0.3192	0.6096	0.4284	0.031*	
C15	0.4312 (3)	0.6068 (5)	0.3723 (2)	0.0276 (8)	
C16	0.4526 (3)	0.5653 (5)	0.3038 (2)	0.0261 (8)	
C17	0.3717 (4)	0.5003 (5)	0.2454 (2)	0.0333 (10)	
H17	0.3853	0.4708	0.1998	0.040*	
C18	0.2703 (3)	0.4794 (5)	0.2550 (2)	0.0287 (9)	
O19	0.5173 (2)	0.6665 (4)	0.42784 (16)	0.0332 (7)	
C20	0.4963 (3)	0.7129 (5)	0.4966 (2)	0.0305 (9)	
H20A	0.5622	0.7404	0.5334	0.037*	
H20B	0.4657	0.6256	0.5172	0.037*	
C21	0.4215 (3)	0.8507 (5)	0.4859 (2)	0.0314 (9)	
O22	0.4065 (2)	0.9443 (4)	0.43373 (18)	0.0378 (7)	
O23	0.3748 (2)	0.8571 (4)	0.54280 (19)	0.0391 (7)	
C24	0.3069 (5)	0.9896 (6)	0.5401 (4)	0.0515 (14)	
H24A	0.2574	0.9958	0.4905	0.077*	
H24B	0.3486	1.0832	0.5496	0.077*	
H24C	0.2692	0.9784	0.5782	0.077*	
O25	0.5533 (2)	0.5945 (4)	0.29926 (17)	0.0372 (7)	
C26	0.5731 (4)	0.5677 (7)	0.2266 (3)	0.0484 (13)	
H26A	0.5261	0.6308	0.1886	0.073*	
H26B	0.5615	0.4594	0.2132	0.073*	
H26C	0.6448	0.5949	0.2294	0.073*	
C27	0.1773 (4)	0.4205 (5)	0.1925 (2)	0.0351 (10)	
H27A	0.1998	0.3353	0.1655	0.042*	
H27B	0.1506	0.5038	0.1563	0.042*	
C28	0.0905 (4)	0.3645 (5)	0.2267 (2)	0.0356 (10)	
H28	0.0194	0.3587	0.1916	0.043*	
C29	0.1124 (4)	0.2580 (5)	0.2867 (3)	0.0355 (10)	
H29A	0.1808	0.2204	0.3062	0.043*	
H29B	0.0588	0.2243	0.3072	0.043*	
O30	0.1772 (6)	0.9369 (8)	0.3617 (4)	0.043 (3)	0.474 (11)

### [4-Methoxy-5-(2-methoxy-2-oxoethoxy)-2-(prop-2-en-1-yl)phenyl](quinolin-8-olato)platinum(II) (I) Atomic displacement parameters (Å^2^)

**Table:**

	*U*^11^	*U*^22^	*U*^33^	*U*^12^	*U*^13^	*U*^23^
Pt1	0.02183 (8)	0.01695 (8)	0.02025 (8)	−0.00247 (5)	0.00636 (6)	−0.00315 (5)
N2	0.0254 (17)	0.0211 (16)	0.0221 (16)	−0.0012 (13)	0.0034 (13)	0.0037 (13)
C3	0.027 (2)	0.0211 (19)	0.031 (2)	−0.0032 (16)	0.0027 (17)	0.0019 (16)
C4	0.024 (2)	0.028 (2)	0.044 (3)	−0.0073 (17)	0.0023 (19)	0.0074 (19)
C5	0.020 (2)	0.031 (2)	0.043 (2)	−0.0008 (17)	0.0088 (18)	0.0131 (19)
C6	0.0229 (19)	0.0227 (19)	0.030 (2)	0.0037 (15)	0.0080 (16)	0.0129 (16)
C7	0.0220 (18)	0.0165 (17)	0.0231 (18)	0.0017 (14)	0.0058 (15)	0.0065 (14)
C8	0.025 (2)	0.027 (2)	0.032 (2)	0.0074 (16)	0.0144 (17)	0.0112 (17)
C9	0.033 (2)	0.024 (2)	0.026 (2)	0.0113 (17)	0.0139 (17)	0.0086 (16)
C10	0.027 (2)	0.0167 (18)	0.0210 (19)	0.0015 (15)	0.0065 (15)	0.0028 (14)
C11	0.0224 (18)	0.0157 (17)	0.0216 (18)	0.0031 (14)	0.0059 (15)	0.0055 (14)
O12	0.0213 (13)	0.0191 (13)	0.0217 (13)	−0.0015 (10)	0.0084 (10)	−0.0020 (10)
C13	0.0231 (19)	0.0185 (18)	0.0234 (19)	0.0023 (14)	0.0063 (15)	0.0007 (14)
C14	0.029 (2)	0.026 (2)	0.026 (2)	0.0003 (16)	0.0114 (17)	−0.0068 (16)
C15	0.026 (2)	0.028 (2)	0.029 (2)	0.0002 (16)	0.0074 (17)	−0.0045 (17)
C16	0.024 (2)	0.031 (2)	0.026 (2)	0.0091 (17)	0.0104 (16)	0.0040 (16)
C17	0.035 (2)	0.040 (3)	0.028 (2)	0.0075 (19)	0.0138 (19)	−0.0071 (18)
C18	0.034 (2)	0.026 (2)	0.028 (2)	0.0000 (17)	0.0120 (18)	−0.0068 (16)
O19	0.0257 (15)	0.0421 (18)	0.0327 (16)	−0.0013 (13)	0.0093 (12)	−0.0056 (13)
C20	0.030 (2)	0.030 (2)	0.030 (2)	−0.0008 (17)	0.0057 (17)	−0.0048 (17)
C21	0.028 (2)	0.033 (2)	0.035 (2)	−0.0085 (18)	0.0109 (18)	−0.0052 (19)
O22	0.0391 (18)	0.0350 (17)	0.0384 (18)	−0.0060 (14)	0.0092 (14)	0.0021 (14)
O23	0.0390 (18)	0.0357 (18)	0.0486 (19)	0.0007 (14)	0.0220 (15)	−0.0002 (15)
C24	0.056 (3)	0.043 (3)	0.065 (4)	0.009 (2)	0.032 (3)	0.004 (2)
O25	0.0225 (15)	0.061 (2)	0.0305 (16)	0.0052 (14)	0.0120 (12)	0.0008 (15)
C26	0.027 (2)	0.089 (4)	0.035 (3)	0.010 (3)	0.017 (2)	0.002 (3)
C27	0.040 (3)	0.035 (2)	0.031 (2)	−0.004 (2)	0.0107 (19)	−0.0076 (19)
C28	0.040 (3)	0.039 (3)	0.031 (2)	−0.016 (2)	0.0144 (19)	−0.0216 (19)
C29	0.036 (2)	0.027 (2)	0.046 (3)	−0.0042 (18)	0.015 (2)	−0.0167 (19)
O30	0.043 (4)	0.033 (4)	0.051 (5)	0.001 (3)	0.008 (3)	−0.009 (3)

### [4-Methoxy-5-(2-methoxy-2-oxoethoxy)-2-(prop-2-en-1-yl)phenyl](quinolin-8-olato)platinum(II) (I) Geometric parameters (Å, º)

**Table:**

Pt1—N2	2.114 (3)	C15—O19	1.395 (5)
Pt1—O12	2.028 (2)	C16—C17	1.399 (6)
Pt1—C13	1.993 (4)	C16—O25	1.372 (5)
Pt1—C28	2.132 (4)	C17—H17	0.9300
Pt1—C29	2.123 (4)	C17—C18	1.402 (6)
N2—C3	1.326 (5)	C18—C27	1.513 (6)
N2—C7	1.377 (5)	O19—C20	1.411 (5)
C3—H3	0.9300	C20—H20A	0.9700
C3—C4	1.401 (6)	C20—H20B	0.9700
C4—H4	0.9300	C20—C21	1.514 (6)
C4—C5	1.369 (6)	C21—O22	1.217 (5)
C5—H5	0.9300	C21—O23	1.341 (5)
C5—C6	1.416 (6)	O23—C24	1.436 (6)
C6—C7	1.420 (5)	C24—H24A	0.9600
C6—C8	1.409 (6)	C24—H24B	0.9600
C7—C11	1.427 (5)	C24—H24C	0.9600
C8—H8	0.9300	O25—C26	1.434 (5)
C8—C9	1.371 (6)	C26—H26A	0.9600
C9—H9	0.9300	C26—H26B	0.9600
C9—C10	1.414 (5)	C26—H26C	0.9600
C10—H10	0.9300	C27—H27A	0.9700
C10—C11	1.381 (5)	C27—H27B	0.9700
C11—O12	1.339 (4)	C27—C28	1.519 (6)
C13—C14	1.401 (5)	C28—H28	0.9800
C13—C18	1.394 (5)	C28—C29	1.391 (7)
C14—H14	0.9300	C29—H29A	0.9300
C14—C15	1.382 (5)	C29—H29B	0.9300
C15—C16	1.396 (5)		
			
N2—Pt1—C28	103.50 (15)	O25—C16—C15	116.0 (4)
N2—Pt1—C29	96.79 (15)	O25—C16—C17	125.1 (4)
O12—Pt1—N2	81.35 (11)	C16—C17—H17	119.7
O12—Pt1—C28	160.78 (15)	C16—C17—C18	120.6 (4)
O12—Pt1—C29	160.82 (15)	C18—C17—H17	119.7
C13—Pt1—N2	174.70 (14)	C13—C18—C17	120.1 (4)
C13—Pt1—O12	93.43 (13)	C13—C18—C27	116.4 (4)
C13—Pt1—C28	81.74 (16)	C17—C18—C27	123.4 (4)
C13—Pt1—C29	87.83 (16)	C15—O19—C20	115.6 (3)
C29—Pt1—C28	38.17 (18)	O19—C20—H20A	109.1
C3—N2—Pt1	131.3 (3)	O19—C20—H20B	109.1
C3—N2—C7	118.8 (3)	O19—C20—C21	112.4 (3)
C7—N2—Pt1	109.8 (2)	H20A—C20—H20B	107.9
N2—C3—H3	118.8	C21—C20—H20A	109.1
N2—C3—C4	122.4 (4)	C21—C20—H20B	109.1
C4—C3—H3	118.8	O22—C21—C20	125.1 (4)
C3—C4—H4	120.0	O22—C21—O23	124.4 (4)
C5—C4—C3	120.0 (4)	O23—C21—C20	110.5 (4)
C5—C4—H4	120.0	C21—O23—C24	114.6 (4)
C4—C5—H5	120.1	O23—C24—H24A	109.5
C4—C5—C6	119.7 (4)	O23—C24—H24B	109.5
C6—C5—H5	120.1	O23—C24—H24C	109.5
C5—C6—C7	117.0 (4)	H24A—C24—H24B	109.5
C8—C6—C5	124.5 (4)	H24A—C24—H24C	109.5
C8—C6—C7	118.5 (4)	H24B—C24—H24C	109.5
N2—C7—C6	122.1 (3)	C16—O25—C26	116.4 (3)
N2—C7—C11	116.6 (3)	O25—C26—H26A	109.5
C6—C7—C11	121.3 (4)	O25—C26—H26B	109.5
C6—C8—H8	120.0	O25—C26—H26C	109.5
C9—C8—C6	119.9 (4)	H26A—C26—H26B	109.5
C9—C8—H8	120.0	H26A—C26—H26C	109.5
C8—C9—H9	119.2	H26B—C26—H26C	109.5
C8—C9—C10	121.5 (4)	C18—C27—H27A	109.7
C10—C9—H9	119.2	C18—C27—H27B	109.7
C9—C10—H10	119.7	C18—C27—C28	110.0 (4)
C11—C10—C9	120.6 (4)	H27A—C27—H27B	108.2
C11—C10—H10	119.7	C28—C27—H27A	109.7
C10—C11—C7	118.0 (3)	C28—C27—H27B	109.7
O12—C11—C7	119.3 (3)	Pt1—C28—H28	116.0
O12—C11—C10	122.7 (3)	C27—C28—Pt1	109.1 (3)
C11—O12—Pt1	112.7 (2)	C27—C28—H28	116.0
C14—C13—Pt1	124.5 (3)	C29—C28—Pt1	70.5 (2)
C18—C13—Pt1	116.8 (3)	C29—C28—C27	120.6 (4)
C18—C13—C14	118.7 (4)	C29—C28—H28	116.0
C13—C14—H14	119.4	Pt1—C29—H29A	108.6
C15—C14—C13	121.2 (4)	Pt1—C29—H29B	90.1
C15—C14—H14	119.4	C28—C29—Pt1	71.3 (2)
C14—C15—C16	120.3 (4)	C28—C29—H29A	120.0
C14—C15—O19	124.7 (3)	C28—C29—H29B	120.0
O19—C15—C16	114.9 (3)	H29A—C29—H29B	120.0
C15—C16—C17	119.0 (4)		
			
Pt1—N2—C3—C4	174.8 (3)	C10—C11—O12—Pt1	178.0 (3)
Pt1—N2—C7—C6	−176.0 (3)	C13—C14—C15—C16	−1.6 (6)
Pt1—N2—C7—C11	4.4 (4)	C13—C14—C15—O19	−179.6 (4)
Pt1—C13—C14—C15	−179.8 (3)	C13—C18—C27—C28	−21.0 (5)
Pt1—C13—C18—C17	−179.3 (3)	C14—C13—C18—C17	−1.0 (6)
Pt1—C13—C18—C27	4.3 (5)	C14—C13—C18—C27	−177.4 (4)
N2—C3—C4—C5	0.5 (6)	C14—C15—C16—C17	0.1 (6)
N2—C7—C11—C10	178.1 (3)	C14—C15—C16—O25	179.2 (4)
N2—C7—C11—O12	−1.6 (5)	C14—C15—O19—C20	−3.5 (6)
C3—N2—C7—C6	0.8 (5)	C15—C16—C17—C18	1.0 (6)
C3—N2—C7—C11	−178.8 (3)	C15—C16—O25—C26	−173.7 (4)
C3—C4—C5—C6	0.7 (6)	C15—O19—C20—C21	−65.7 (5)
C4—C5—C6—C7	−1.1 (6)	C16—C15—O19—C20	178.4 (4)
C4—C5—C6—C8	178.8 (4)	C16—C17—C18—C13	−0.5 (6)
C5—C6—C7—N2	0.4 (5)	C16—C17—C18—C27	175.6 (4)
C5—C6—C7—C11	179.9 (3)	C17—C16—O25—C26	5.4 (6)
C5—C6—C8—C9	−178.8 (4)	C17—C18—C27—C28	162.8 (4)
C6—C7—C11—C10	−1.5 (5)	C18—C13—C14—C15	2.0 (6)
C6—C7—C11—O12	178.8 (3)	C18—C27—C28—Pt1	26.3 (5)
C6—C8—C9—C10	−0.7 (6)	C18—C27—C28—C29	−51.9 (5)
C7—N2—C3—C4	−1.2 (6)	O19—C15—C16—C17	178.3 (4)
C7—C6—C8—C9	1.1 (5)	O19—C15—C16—O25	−2.6 (5)
C7—C11—O12—Pt1	−2.3 (4)	O19—C20—C21—O22	−22.6 (6)
C8—C6—C7—N2	−179.6 (3)	O19—C20—C21—O23	158.3 (3)
C8—C6—C7—C11	0.0 (5)	C20—C21—O23—C24	176.5 (4)
C8—C9—C10—C11	−0.8 (6)	O22—C21—O23—C24	−2.6 (6)
C9—C10—C11—C7	1.9 (5)	O25—C16—C17—C18	−178.1 (4)
C9—C10—C11—O12	−178.4 (3)	C27—C28—C29—Pt1	101.2 (4)

### [4-Methoxy-5-(2-methoxy-2-oxoethoxy)-2-(prop-2-en-1-yl)phenyl](quinolin-8-olato)platinum(II) (I) Hydrogen-bond geometry (Å, º)

*Cg*1 is the centroid of ring C6–C11.

**Table:**

*D*—H···*A*	*D*—H	H···*A*	*D*···*A*	*D*—H···*A*
C27—H27*A*···*Cg*1^i^	0.97	2.81	3.465 (4)	125

Symmetry code: (i) −*x*, *y*−1/2, −*z*+1/2.

### [4-Methoxy-5-(2-oxo-2-propoxyethoxy)-2-(prop-2-en-1-yl)phenyl](quinoline-2-carboxylato)platinum(II) (II) Crystal data

**Table:**

[Pt(C_15_H_19_O_4_)(C_10_H6NO_2_)]	*F*(000) = 1232
*M**_r_* = 630.55	*D*_x_ = 1.910 Mg m^−^^3^
Monoclinic, *P*2_1_/*n*	Mo *K*α radiation, λ = 0.71073 Å
*a* = 8.2857 (4) Å	Cell parameters from 8290 reflections
*b* = 18.5001 (9) Å	θ = 2.9–29.0°
*c* = 14.6282 (7) Å	µ = 6.44 mm^−^^1^
β = 102.014 (5)°	*T* = 100 K
*V* = 2193.18 (19) Å^3^	Block, orange
*Z* = 4	0.4 × 0.4 × 0.3 mm

### [4-Methoxy-5-(2-oxo-2-propoxyethoxy)-2-(prop-2-en-1-yl)phenyl](quinoline-2-carboxylato)platinum(II) (II) Data collection

**Table:**

SuperNova, Single source at offset, Eos diffractometer	5366 independent reflections
Radiation source: micro-focus sealed X-ray tube, SuperNova (Mo) X-ray Source	4652 reflections with *I* > 2σ(*I*)
Mirror monochromator	*R*_int_ = 0.057
Detector resolution: 15.9631 pixels mm^-1^	θ_max_ = 29.1°, θ_min_ = 2.6°
ω scans	*h* = −11→11
Absorption correction: multi-scan (CrysAlisPro; Rigaku OD, 2022)	*k* = −25→24
*T*_min_ = 0.669, *T*_max_ = 1.000	*l* = −19→19
23965 measured reflections	

### [4-Methoxy-5-(2-oxo-2-propoxyethoxy)-2-(prop-2-en-1-yl)phenyl](quinoline-2-carboxylato)platinum(II) (II) Refinement

**Table:**

Refinement on *F*^2^	0 restraints
Least-squares matrix: full	Hydrogen site location: inferred from neighbouring sites
*R*[*F*^2^ > 2σ(*F*^2^)] = 0.033	H-atom parameters constrained
*wR*(*F*^2^) = 0.070	*w* = 1/[σ^2^(*F*_o_^2^) + (0.0204*P*)^2^ + 5.0718*P*] where *P* = (*F*_o_^2^ + 2*F*_c_^2^)/3
*S* = 1.05	(Δ/σ)_max_ = 0.003
5366 reflections	Δρ_max_ = 2.16 e Å^−^^3^
300 parameters	Δρ_min_ = −1.73 e Å^−^^3^

### [4-Methoxy-5-(2-oxo-2-propoxyethoxy)-2-(prop-2-en-1-yl)phenyl](quinoline-2-carboxylato)platinum(II) (II) Special details

**Table:**

Geometry. All esds (except the esd in the dihedral angle between two l.s. planes) are estimated using the full covariance matrix. The cell esds are taken into account individually in the estimation of esds in distances, angles and torsion angles; correlations between esds in cell parameters are only used when they are defined by crystal symmetry. An approximate (isotropic) treatment of cell esds is used for estimating esds involving l.s. planes.

### [4-Methoxy-5-(2-oxo-2-propoxyethoxy)-2-(prop-2-en-1-yl)phenyl](quinoline-2-carboxylato)platinum(II) (II) Fractional atomic coordinates and isotropic or equivalent isotropic displacement parameters (Å^2^)

**Table:**

	*x*	*y*	*z*	*U*_iso_*/*U*_eq_	
Pt1	−0.05560 (2)	0.61639 (2)	0.09345 (2)	0.01722 (6)	
C2	−0.3155 (5)	0.6274 (2)	0.0693 (3)	0.0219 (9)	
H2A	−0.3776	0.6082	0.0108	0.026*	
H2B	−0.3664	0.6180	0.1221	0.026*	
C3	−0.2450 (5)	0.6967 (2)	0.0656 (3)	0.0204 (9)	
H3	−0.2631	0.7194	0.0038	0.024*	
C4	−0.2226 (6)	0.7476 (2)	0.1480 (3)	0.0233 (10)	
H4A	−0.3286	0.7671	0.1538	0.028*	
H4B	−0.1520	0.7875	0.1385	0.028*	
C5	−0.1454 (5)	0.7072 (2)	0.2364 (3)	0.0183 (9)	
C6	−0.0619 (5)	0.6442 (2)	0.2243 (3)	0.0164 (8)	
C7	0.0167 (5)	0.6048 (2)	0.3027 (3)	0.0195 (9)	
H7	0.0717	0.5621	0.2947	0.023*	
C8	0.0134 (5)	0.6286 (2)	0.3922 (3)	0.0191 (9)	
C9	−0.0748 (5)	0.6916 (2)	0.4044 (3)	0.0180 (9)	
C10	−0.1524 (5)	0.7309 (2)	0.3268 (3)	0.0194 (9)	
H10	−0.2091	0.7730	0.3347	0.023*	
O11	−0.0792 (4)	0.70854 (15)	0.4954 (2)	0.0207 (6)	
C12	−0.1635 (6)	0.7729 (2)	0.5106 (3)	0.0248 (10)	
H12A	−0.1152	0.8133	0.4850	0.037*	
H12B	−0.1546	0.7800	0.5765	0.037*	
H12C	−0.2777	0.7689	0.4805	0.037*	
O13	0.0916 (4)	0.59562 (16)	0.4739 (2)	0.0228 (7)	
C14	0.1812 (6)	0.5321 (2)	0.4651 (3)	0.0219 (9)	
H14A	0.2499	0.5392	0.4196	0.026*	
H14B	0.1061	0.4924	0.4442	0.026*	
C15	0.2864 (5)	0.5154 (2)	0.5592 (3)	0.0204 (9)	
O16	0.3081 (4)	0.55414 (18)	0.6260 (2)	0.0335 (8)	
O17	0.3562 (4)	0.45000 (16)	0.5567 (2)	0.0259 (7)	
C18	0.4476 (6)	0.4221 (2)	0.6464 (3)	0.0262 (10)	
H18A	0.3748	0.4161	0.6899	0.031*	
H18B	0.5357	0.4550	0.6736	0.031*	
C19	0.5177 (6)	0.3497 (2)	0.6249 (3)	0.0298 (11)	
H19A	0.6013	0.3571	0.5883	0.036*	
H19B	0.4307	0.3202	0.5886	0.036*	
C20	0.5934 (6)	0.3109 (3)	0.7157 (3)	0.0348 (12)	
H20A	0.6450	0.2670	0.7016	0.052*	
H20B	0.5084	0.2997	0.7491	0.052*	
H20C	0.6744	0.3415	0.7533	0.052*	
N21	0.0072 (4)	0.59004 (18)	−0.0416 (2)	0.0165 (7)	
C22	−0.0717 (6)	0.6046 (2)	−0.1330 (3)	0.0210 (9)	
C23	−0.2406 (6)	0.6240 (2)	−0.1543 (3)	0.0229 (10)	
H23	−0.2981	0.6286	−0.1063	0.027*	
C24	−0.3208 (7)	0.6363 (2)	−0.2454 (3)	0.0295 (11)	
H24	−0.4326	0.6477	−0.2585	0.035*	
C25	−0.2346 (7)	0.6316 (2)	−0.3190 (3)	0.0301 (11)	
H25	−0.2888	0.6408	−0.3802	0.036*	
C26	−0.0716 (7)	0.6135 (2)	−0.2999 (3)	0.0272 (11)	
H26	−0.0144	0.6117	−0.3482	0.033*	
C27	0.0111 (6)	0.5975 (2)	−0.2081 (3)	0.0225 (10)	
C28	0.1770 (6)	0.5718 (2)	−0.1873 (3)	0.0262 (10)	
H28	0.2359	0.5675	−0.2346	0.031*	
C29	0.2488 (6)	0.5537 (2)	−0.0978 (3)	0.0228 (10)	
H29	0.3547	0.5344	−0.0840	0.027*	
C30	0.1604 (5)	0.5647 (2)	−0.0265 (3)	0.0201 (9)	
C31	0.2456 (5)	0.5489 (2)	0.0731 (3)	0.0194 (9)	
O32	0.3826 (4)	0.52108 (16)	0.0902 (2)	0.0251 (7)	
O33	0.1638 (3)	0.56700 (16)	0.13703 (19)	0.0205 (6)	

### [4-Methoxy-5-(2-oxo-2-propoxyethoxy)-2-(prop-2-en-1-yl)phenyl](quinoline-2-carboxylato)platinum(II) (II) Atomic displacement parameters (Å^2^)

**Table:**

	*U*^11^	*U*^22^	*U*^33^	*U*^12^	*U*^13^	*U*^23^
Pt1	0.01864 (10)	0.01974 (10)	0.01431 (10)	0.00199 (6)	0.00582 (7)	0.00078 (6)
C2	0.019 (2)	0.024 (2)	0.023 (2)	0.0026 (18)	0.0041 (19)	−0.0017 (18)
C3	0.019 (2)	0.026 (2)	0.016 (2)	0.0077 (18)	0.0024 (17)	0.0028 (17)
C4	0.027 (2)	0.015 (2)	0.026 (2)	0.0021 (18)	0.002 (2)	−0.0026 (17)
C5	0.019 (2)	0.0157 (19)	0.019 (2)	−0.0028 (17)	0.0017 (17)	0.0021 (16)
C6	0.017 (2)	0.0179 (19)	0.016 (2)	−0.0012 (17)	0.0053 (16)	0.0001 (16)
C7	0.018 (2)	0.019 (2)	0.023 (2)	0.0052 (17)	0.0076 (18)	0.0005 (17)
C8	0.020 (2)	0.023 (2)	0.016 (2)	−0.0004 (18)	0.0055 (17)	0.0013 (17)
C9	0.019 (2)	0.0162 (19)	0.020 (2)	−0.0033 (17)	0.0058 (18)	−0.0026 (16)
C10	0.021 (2)	0.0148 (19)	0.022 (2)	0.0019 (17)	0.0044 (18)	−0.0035 (17)
O11	0.0299 (17)	0.0166 (14)	0.0164 (15)	0.0046 (13)	0.0064 (13)	−0.0026 (12)
C12	0.026 (2)	0.023 (2)	0.025 (2)	0.0059 (19)	0.005 (2)	−0.0056 (19)
O13	0.0315 (18)	0.0233 (15)	0.0135 (15)	0.0115 (14)	0.0043 (13)	0.0014 (12)
C14	0.028 (2)	0.021 (2)	0.019 (2)	0.0087 (19)	0.0093 (19)	0.0023 (17)
C15	0.023 (2)	0.022 (2)	0.019 (2)	0.0069 (18)	0.0114 (18)	0.0034 (17)
O16	0.045 (2)	0.0364 (19)	0.0175 (17)	0.0176 (17)	0.0040 (15)	−0.0044 (14)
O17	0.0318 (18)	0.0206 (16)	0.0248 (17)	0.0071 (14)	0.0050 (14)	0.0029 (13)
C18	0.029 (3)	0.030 (2)	0.021 (2)	0.007 (2)	0.0062 (19)	0.0090 (19)
C19	0.040 (3)	0.016 (2)	0.034 (3)	0.000 (2)	0.009 (2)	0.0036 (19)
C20	0.037 (3)	0.027 (3)	0.037 (3)	−0.002 (2)	0.001 (2)	0.007 (2)
N21	0.0200 (18)	0.0166 (17)	0.0143 (17)	0.0002 (14)	0.0067 (14)	0.0017 (13)
C22	0.030 (3)	0.0138 (19)	0.020 (2)	−0.0051 (18)	0.0066 (19)	−0.0021 (16)
C23	0.030 (3)	0.021 (2)	0.018 (2)	0.0016 (19)	0.007 (2)	−0.0034 (17)
C24	0.037 (3)	0.029 (2)	0.020 (2)	0.003 (2)	−0.002 (2)	0.0030 (19)
C25	0.050 (3)	0.020 (2)	0.017 (2)	0.002 (2)	0.001 (2)	0.0040 (18)
C26	0.044 (3)	0.019 (2)	0.020 (2)	−0.009 (2)	0.010 (2)	−0.0025 (17)
C27	0.036 (3)	0.0148 (19)	0.020 (2)	−0.0104 (19)	0.013 (2)	−0.0057 (17)
C28	0.037 (3)	0.023 (2)	0.024 (2)	−0.005 (2)	0.019 (2)	−0.0091 (19)
C29	0.023 (2)	0.021 (2)	0.027 (2)	−0.0051 (18)	0.011 (2)	−0.0051 (18)
C30	0.024 (2)	0.0136 (19)	0.025 (2)	−0.0048 (17)	0.0101 (19)	−0.0045 (17)
C31	0.021 (2)	0.017 (2)	0.021 (2)	−0.0026 (17)	0.0062 (18)	−0.0045 (17)
O32	0.0193 (16)	0.0266 (17)	0.0302 (18)	0.0015 (14)	0.0067 (14)	−0.0010 (14)
O33	0.0195 (16)	0.0270 (16)	0.0147 (15)	0.0020 (13)	0.0034 (12)	−0.0008 (12)

### [4-Methoxy-5-(2-oxo-2-propoxyethoxy)-2-(prop-2-en-1-yl)phenyl](quinoline-2-carboxylato)platinum(II) (II) Geometric parameters (Å, º)

**Table:**

Pt1—C2	2.118 (4)	C15—O17	1.345 (5)
Pt1—C3	2.138 (4)	O17—C18	1.466 (5)
Pt1—C6	1.993 (4)	C18—H18A	0.9700
Pt1—N21	2.200 (3)	C18—H18B	0.9700
Pt1—O33	2.016 (3)	C18—C19	1.519 (6)
C2—H2A	0.9700	C19—H19A	0.9700
C2—H2B	0.9700	C19—H19B	0.9700
C2—C3	1.413 (6)	C19—C20	1.524 (6)
C3—H3	0.9800	C20—H20A	0.9600
C3—C4	1.511 (6)	C20—H20B	0.9600
C4—H4A	0.9700	C20—H20C	0.9600
C4—H4B	0.9700	N21—C22	1.387 (5)
C4—C5	1.515 (6)	N21—C30	1.328 (5)
C5—C6	1.385 (6)	C22—C23	1.415 (6)
C5—C10	1.406 (6)	C22—C27	1.417 (6)
C6—C7	1.400 (6)	C23—H23	0.9300
C7—H7	0.9300	C23—C24	1.378 (6)
C7—C8	1.387 (6)	C24—H24	0.9300
C8—C9	1.407 (6)	C24—C25	1.413 (7)
C8—O13	1.378 (5)	C25—H25	0.9300
C9—C10	1.388 (6)	C25—C26	1.362 (7)
C9—O11	1.375 (5)	C26—H26	0.9300
C10—H10	0.9300	C26—C27	1.405 (6)
O11—C12	1.422 (5)	C27—C28	1.425 (7)
C12—H12A	0.9600	C28—H28	0.9300
C12—H12B	0.9600	C28—C29	1.363 (6)
C12—H12C	0.9600	C29—H29	0.9300
O13—C14	1.411 (5)	C29—C30	1.407 (6)
C14—H14A	0.9700	C30—C31	1.510 (6)
C14—H14B	0.9700	C31—O32	1.224 (5)
C14—C15	1.500 (6)	C31—O33	1.307 (5)
C15—O16	1.195 (5)		
			
C2—Pt1—C3	38.79 (16)	C15—C14—H14A	110.2
C2—Pt1—N21	107.11 (15)	C15—C14—H14B	110.2
C3—Pt1—N21	106.53 (14)	O16—C15—C14	125.9 (4)
C6—Pt1—C2	84.62 (17)	O16—C15—O17	124.7 (4)
C6—Pt1—C3	80.72 (16)	O17—C15—C14	109.4 (4)
C6—Pt1—N21	167.94 (15)	C15—O17—C18	115.9 (3)
C6—Pt1—O33	90.88 (14)	O17—C18—H18A	110.6
O33—Pt1—C2	156.21 (14)	O17—C18—H18B	110.6
O33—Pt1—C3	162.41 (14)	O17—C18—C19	105.9 (4)
O33—Pt1—N21	79.46 (12)	H18A—C18—H18B	108.7
Pt1—C2—H2A	116.5	C19—C18—H18A	110.6
Pt1—C2—H2B	116.5	C19—C18—H18B	110.6
H2A—C2—H2B	113.5	C18—C19—H19A	109.7
C3—C2—Pt1	71.3 (2)	C18—C19—H19B	109.7
C3—C2—H2A	116.5	C18—C19—C20	109.9 (4)
C3—C2—H2B	116.5	H19A—C19—H19B	108.2
Pt1—C3—H3	116.0	C20—C19—H19A	109.7
C2—C3—Pt1	69.9 (2)	C20—C19—H19B	109.7
C2—C3—H3	116.0	C19—C20—H20A	109.5
C2—C3—C4	121.2 (4)	C19—C20—H20B	109.5
C4—C3—Pt1	108.5 (3)	C19—C20—H20C	109.5
C4—C3—H3	116.0	H20A—C20—H20B	109.5
C3—C4—H4A	109.8	H20A—C20—H20C	109.5
C3—C4—H4B	109.8	H20B—C20—H20C	109.5
C3—C4—C5	109.5 (3)	C22—N21—Pt1	132.1 (3)
H4A—C4—H4B	108.2	C30—N21—Pt1	109.0 (3)
C5—C4—H4A	109.8	C30—N21—C22	118.1 (4)
C5—C4—H4B	109.8	N21—C22—C23	120.5 (4)
C6—C5—C4	116.1 (4)	N21—C22—C27	121.4 (4)
C6—C5—C10	120.1 (4)	C23—C22—C27	118.0 (4)
C10—C5—C4	123.8 (4)	C22—C23—H23	119.7
C5—C6—Pt1	117.0 (3)	C24—C23—C22	120.6 (4)
C5—C6—C7	119.6 (4)	C24—C23—H23	119.7
C7—C6—Pt1	123.5 (3)	C23—C24—H24	119.7
C6—C7—H7	119.7	C23—C24—C25	120.5 (5)
C8—C7—C6	120.7 (4)	C25—C24—H24	119.7
C8—C7—H7	119.7	C24—C25—H25	120.2
C7—C8—C9	119.8 (4)	C26—C25—C24	119.7 (4)
O13—C8—C7	125.5 (4)	C26—C25—H25	120.2
O13—C8—C9	114.8 (4)	C25—C26—H26	119.5
C10—C9—C8	119.6 (4)	C25—C26—C27	120.9 (5)
O11—C9—C8	115.6 (4)	C27—C26—H26	119.5
O11—C9—C10	124.8 (4)	C22—C27—C28	117.8 (4)
C5—C10—H10	119.9	C26—C27—C22	120.1 (5)
C9—C10—C5	120.2 (4)	C26—C27—C28	122.1 (4)
C9—C10—H10	119.9	C27—C28—H28	120.1
C9—O11—C12	117.1 (3)	C29—C28—C27	119.7 (4)
O11—C12—H12A	109.5	C29—C28—H28	120.1
O11—C12—H12B	109.5	C28—C29—H29	120.5
O11—C12—H12C	109.5	C28—C29—C30	119.1 (4)
H12A—C12—H12B	109.5	C30—C29—H29	120.5
H12A—C12—H12C	109.5	N21—C30—C29	123.7 (4)
H12B—C12—H12C	109.5	N21—C30—C31	117.8 (4)
C8—O13—C14	116.8 (3)	C29—C30—C31	118.5 (4)
O13—C14—H14A	110.2	O32—C31—C30	120.3 (4)
O13—C14—H14B	110.2	O32—C31—O33	124.0 (4)
O13—C14—C15	107.7 (3)	O33—C31—C30	115.7 (4)
H14A—C14—H14B	108.5	C31—O33—Pt1	117.2 (3)
			
Pt1—C2—C3—C4	−100.0 (4)	O13—C14—C15—O17	171.5 (3)
Pt1—C3—C4—C5	−30.0 (4)	C14—C15—O17—C18	−173.1 (4)
Pt1—C6—C7—C8	177.3 (3)	C15—O17—C18—C19	−178.4 (4)
Pt1—N21—C22—C23	−18.3 (6)	O16—C15—O17—C18	8.0 (6)
Pt1—N21—C22—C27	164.5 (3)	O17—C18—C19—C20	−171.5 (4)
Pt1—N21—C30—C29	−169.3 (3)	N21—C22—C23—C24	−177.9 (4)
Pt1—N21—C30—C31	8.9 (4)	N21—C22—C27—C26	−179.2 (4)
C2—C3—C4—C5	47.2 (5)	N21—C22—C27—C28	2.6 (6)
C3—C4—C5—C6	21.0 (5)	N21—C30—C31—O32	175.2 (4)
C3—C4—C5—C10	−160.3 (4)	N21—C30—C31—O33	−5.0 (5)
C4—C5—C6—Pt1	0.0 (5)	C22—N21—C30—C29	1.9 (6)
C4—C5—C6—C7	178.3 (4)	C22—N21—C30—C31	−180.0 (3)
C4—C5—C10—C9	−178.3 (4)	C22—C23—C24—C25	−1.8 (7)
C5—C6—C7—C8	−0.8 (6)	C22—C27—C28—C29	1.4 (6)
C6—C5—C10—C9	0.4 (6)	C23—C22—C27—C26	3.5 (6)
C6—C7—C8—C9	2.4 (6)	C23—C22—C27—C28	−174.7 (4)
C6—C7—C8—O13	−177.4 (4)	C23—C24—C25—C26	1.2 (7)
C7—C8—C9—C10	−2.5 (6)	C24—C25—C26—C27	1.7 (7)
C7—C8—C9—O11	176.3 (4)	C25—C26—C27—C22	−4.1 (6)
C7—C8—O13—C14	−0.4 (6)	C25—C26—C27—C28	174.0 (4)
C8—C9—C10—C5	1.2 (6)	C26—C27—C28—C29	−176.7 (4)
C8—C9—O11—C12	178.3 (4)	C27—C22—C23—C24	−0.6 (6)
C8—O13—C14—C15	167.0 (4)	C27—C28—C29—C30	−3.6 (6)
C9—C8—O13—C14	179.8 (4)	C28—C29—C30—N21	2.0 (6)
C10—C5—C6—Pt1	−178.8 (3)	C28—C29—C30—C31	−176.1 (4)
C10—C5—C6—C7	−0.5 (6)	C29—C30—C31—O32	−6.5 (6)
C10—C9—O11—C12	−2.9 (6)	C29—C30—C31—O33	173.3 (4)
O11—C9—C10—C5	−177.5 (4)	C30—N21—C22—C23	173.0 (4)
O13—C8—C9—C10	177.3 (4)	C30—N21—C22—C27	−4.2 (6)
O13—C8—C9—O11	−4.0 (5)	C30—C31—O33—Pt1	−2.6 (4)
O13—C14—C15—O16	−9.7 (6)	O32—C31—O33—Pt1	177.2 (3)

### [4-Methoxy-5-(2-oxo-2-propoxyethoxy)-2-(prop-2-en-1-yl)phenyl](quinoline-2-carboxylato)platinum(II) (II) Hydrogen-bond geometry (Å, º)

*Cg*1 and *Cg*2 are the centroids of rings C5–C10 and C22–C27, respectively.

**Table:**

*D*—H···*A*	*D*—H	H···*A*	*D*···*A*	*D*—H···*A*
C20—H20*C*···O33^i^	0.96	2.52	3.462 (6)	168
C28—H28···O16^ii^	0.93	2.26	3.159 (5)	164
C29—H29···O32^iii^	0.93	2.43	3.334 (6)	166
C18—H18*A*···*Cg*1^iv^	0.97	2.97	3.711 (5)	134
C20—H20*A*···*Cg*2^v^	0.96	2.78	3.605 (6)	144

Symmetry codes: (i) −*x*+1, −*y*+1, −*z*+1; (ii) *x*, *y*, *z*−1; (iii) −*x*+1, −*y*+1, −*z*; (iv) −*x*, −*y*+1, −*z*+1; (v) −*x*+1/2, *y*−1/2, −*z*+1/2.

### Chlorido[4-methoxy-5-(2-oxo-2-propoxyethoxy)-2-(prop-2-en-1-yl)phenyl]\ (quinoline)platinum(II) (III) Crystal data

**Table:**

[Pt(C_15_H_19_O_4_)Cl(C_9_H_7_N)]	*F*(000) = 1216
*M**_r_* = 623.00	*D*_x_ = 1.833 Mg m^−^^3^
Monoclinic, *P*2_1_/*c*	Mo *K*α radiation, λ = 0.71073 Å
*a* = 14.576 (2) Å	Cell parameters from 3865 reflections
*b* = 11.0945 (9) Å	θ = 2.9–29.0°
*c* = 15.700 (2) Å	µ = 6.36 mm^−^^1^
β = 117.197 (18)°	*T* = 114 K
*V* = 2258.1 (6) Å^3^	Block, colourless
*Z* = 4	0.27 × 0.2 × 0.16 mm

### Chlorido[4-methoxy-5-(2-oxo-2-propoxyethoxy)-2-(prop-2-en-1-yl)phenyl]\ (quinoline)platinum(II) (III) Data collection

**Table:**

SuperNova, Single source at offset, Eos diffractometer	4603 independent reflections
Radiation source: SuperNova (Mo) X-ray Source	3885 reflections with *I* > 2σ(*I*)
Mirror monochromator	*R*_int_ = 0.028
Detector resolution: 15.9631 pixels mm^-1^	θ_max_ = 26.4°, θ_min_ = 2.4°
ω scans	*h* = −9→18
Absorption correction: multi-scan (CrysAlisPro; Rigaku OD, 2022)	*k* = −12→13
*T*_min_ = 0.579, *T*_max_ = 1.000	*l* = −19→19
8975 measured reflections	

### Chlorido[4-methoxy-5-(2-oxo-2-propoxyethoxy)-2-(prop-2-en-1-yl)phenyl]\ (quinoline)platinum(II) (III) Refinement

**Table:**

Refinement on *F*^2^	Primary atom site location: structure-invariant direct methods
Least-squares matrix: full	Hydrogen site location: inferred from neighbouring sites
*R*[*F*^2^ > 2σ(*F*^2^)] = 0.030	H-atom parameters constrained
*wR*(*F*^2^) = 0.056	*w* = 1/[σ^2^(*F*_o_^2^) + (0.0148*P*)^2^] where *P* = (*F*_o_^2^ + 2*F*_c_^2^)/3
*S* = 1.04	(Δ/σ)_max_ = 0.001
4603 reflections	Δρ_max_ = 0.85 e Å^−^^3^
292 parameters	Δρ_min_ = −1.07 e Å^−^^3^
288 restraints	

### Chlorido[4-methoxy-5-(2-oxo-2-propoxyethoxy)-2-(prop-2-en-1-yl)phenyl]\ (quinoline)platinum(II) (III) Special details

**Table:**

Geometry. All esds (except the esd in the dihedral angle between two l.s. planes) are estimated using the full covariance matrix. The cell esds are taken into account individually in the estimation of esds in distances, angles and torsion angles; correlations between esds in cell parameters are only used when they are defined by crystal symmetry. An approximate (isotropic) treatment of cell esds is used for estimating esds involving l.s. planes.

### Chlorido[4-methoxy-5-(2-oxo-2-propoxyethoxy)-2-(prop-2-en-1-yl)phenyl]\ (quinoline)platinum(II) (III) Fractional atomic coordinates and isotropic or equivalent isotropic displacement parameters (Å^2^)

**Table:**

	*x*	*y*	*z*	*U*_iso_*/*U*_eq_	Occ. (<1)
Pt1A	0.48946 (7)	0.89396 (12)	0.13147 (5)	0.02532 (15)	0.928 (7)
Pt1B	0.4763 (6)	0.8600 (18)	0.1469 (11)	0.028 (2)	0.072 (7)
Cl2	0.38195 (9)	0.97795 (9)	0.18873 (8)	0.0360 (3)	
N3	0.6140 (3)	0.9769 (3)	0.2531 (2)	0.0344 (9)	
C4	0.6343 (3)	1.0901 (3)	0.2431 (3)	0.0329 (10)	
H4	0.6028	1.1231	0.1803	0.039*	
C5	0.6988 (3)	1.1655 (4)	0.3182 (3)	0.0340 (10)	
H5	0.7100	1.2471	0.3072	0.041*	
C6	0.7452 (3)	1.1163 (4)	0.4087 (3)	0.0355 (11)	
H6	0.7887	1.1645	0.4620	0.043*	
C7	0.7282 (3)	0.9938 (3)	0.4222 (3)	0.0257 (9)	
C8	0.7764 (3)	0.9375 (4)	0.5128 (3)	0.0389 (11)	
H8	0.8211	0.9833	0.5672	0.047*	
C9	0.7601 (4)	0.8201 (4)	0.5239 (3)	0.0437 (12)	
H9	0.7944	0.7827	0.5850	0.052*	
C10	0.6917 (3)	0.7542 (4)	0.4435 (3)	0.0370 (11)	
H10	0.6798	0.6718	0.4516	0.044*	
C11	0.6417 (3)	0.8035 (4)	0.3544 (3)	0.0327 (10)	
H11	0.5950	0.7565	0.3018	0.039*	
C12	0.6604 (3)	0.9264 (4)	0.3413 (3)	0.0285 (9)	
C13	0.5817 (3)	0.8736 (4)	0.0623 (3)	0.0350 (11)	
H13C	0.5462	0.8797	−0.0084	0.042*	0.928 (7)
H13D	0.6496	0.9145	0.0922	0.042*	0.928 (7)
H13A	0.5452	0.8820	−0.0082	0.042*	0.072 (7)
H13B	0.6476	0.9184	0.0940	0.042*	0.072 (7)
C14	0.5759 (3)	0.7628 (4)	0.1010 (3)	0.0329 (10)	
H14A	0.6406	0.7350	0.1570	0.039*	0.928 (7)
H14	0.6423	0.7337	0.1542	0.039*	0.072 (7)
C15	0.5035 (3)	0.6634 (4)	0.0397 (3)	0.0346 (10)	
H15A	0.5298	0.6288	−0.0030	0.042*	
H15B	0.5005	0.5982	0.0814	0.042*	
C16	0.3968 (3)	0.7144 (3)	−0.0195 (3)	0.0280 (9)	
C17	0.3226 (3)	0.6564 (4)	−0.1006 (3)	0.0300 (10)	
H17	0.3400	0.5851	−0.1234	0.036*	
C18	0.2236 (3)	0.7023 (4)	−0.1478 (3)	0.0332 (10)	
C19	0.2006 (3)	0.8102 (4)	−0.1163 (3)	0.0313 (10)	
C20	0.2743 (3)	0.8681 (4)	−0.0358 (3)	0.0293 (10)	
H20	0.2569	0.9412	−0.0152	0.035*	
C21	0.3737 (3)	0.8207 (4)	0.0156 (3)	0.0271 (9)	
O22	0.1425 (2)	0.6476 (3)	−0.2244 (2)	0.0414 (8)	
C23	0.1632 (4)	0.5393 (4)	−0.2615 (3)	0.0449 (13)	
H23A	0.0989	0.5085	−0.3134	0.067*	
H23B	0.1921	0.4790	−0.2104	0.067*	
H23C	0.2127	0.5560	−0.2862	0.067*	
O24	0.1004 (2)	0.8561 (3)	−0.1605 (2)	0.0408 (8)	
C25	0.0681 (3)	0.8996 (4)	−0.2554 (3)	0.0401 (11)	
H25A	−0.0078	0.8928	−0.2920	0.048*	
H25B	0.0988	0.8486	−0.2875	0.048*	
C26	0.0989 (3)	1.0276 (4)	−0.2567 (3)	0.0400 (11)	
O27	0.1496 (3)	1.0896 (3)	−0.1889 (2)	0.0551 (10)	
O28	0.0592 (2)	1.0647 (3)	−0.3482 (2)	0.0442 (8)	
C29	0.0798 (4)	1.1888 (4)	−0.3643 (3)	0.0493 (13)	
H29A	0.1551	1.2023	−0.3376	0.059*	
H29B	0.0521	1.2452	−0.3329	0.059*	
C30	0.0280 (4)	1.2087 (5)	−0.4703 (4)	0.0563 (14)	
H30A	−0.0463	1.1898	−0.4964	0.068*	
H30B	0.0580	1.1533	−0.5004	0.068*	
C31	0.0407 (5)	1.3383 (5)	−0.4953 (4)	0.082 (2)	
H31A	0.0183	1.3446	−0.5642	0.123*	
H31B	0.1133	1.3619	−0.4599	0.123*	
H31C	−0.0015	1.3917	−0.4777	0.123*	

### Chlorido[4-methoxy-5-(2-oxo-2-propoxyethoxy)-2-(prop-2-en-1-yl)phenyl]\ (quinoline)platinum(II) (III) Atomic displacement parameters (Å^2^)

**Table:**

	*U*^11^	*U*^22^	*U*^33^	*U*^12^	*U*^13^	*U*^23^
Pt1A	0.03098 (18)	0.0218 (3)	0.02215 (15)	−0.00439 (19)	0.01123 (11)	−0.00014 (15)
Pt1B	0.0265 (18)	0.025 (4)	0.034 (3)	−0.0041 (19)	0.0147 (16)	−0.008 (3)
Cl2	0.0431 (7)	0.0342 (6)	0.0336 (6)	−0.0016 (5)	0.0199 (5)	−0.0052 (5)
N3	0.043 (2)	0.0285 (18)	0.0320 (19)	−0.0023 (17)	0.0173 (17)	0.0005 (15)
C4	0.041 (3)	0.022 (2)	0.037 (2)	0.001 (2)	0.019 (2)	−0.0007 (18)
C5	0.046 (3)	0.024 (2)	0.041 (2)	−0.006 (2)	0.027 (2)	−0.0057 (19)
C6	0.039 (3)	0.033 (2)	0.040 (2)	−0.008 (2)	0.024 (2)	−0.0127 (19)
C7	0.028 (2)	0.024 (2)	0.028 (2)	−0.0006 (18)	0.0148 (18)	−0.0030 (17)
C8	0.036 (3)	0.042 (2)	0.034 (2)	0.002 (2)	0.012 (2)	0.001 (2)
C9	0.046 (3)	0.044 (3)	0.036 (3)	0.006 (2)	0.014 (2)	0.005 (2)
C10	0.044 (3)	0.035 (2)	0.034 (2)	0.000 (2)	0.019 (2)	0.0068 (19)
C11	0.039 (3)	0.030 (2)	0.031 (2)	−0.004 (2)	0.017 (2)	0.0006 (18)
C12	0.027 (2)	0.035 (2)	0.028 (2)	0.0028 (18)	0.0154 (18)	0.0003 (17)
C13	0.032 (2)	0.041 (2)	0.029 (2)	0.000 (2)	0.011 (2)	−0.006 (2)
C14	0.029 (2)	0.035 (2)	0.030 (2)	0.0054 (19)	0.010 (2)	−0.0046 (19)
C15	0.043 (3)	0.026 (2)	0.033 (2)	0.000 (2)	0.015 (2)	−0.0003 (19)
C16	0.032 (2)	0.023 (2)	0.030 (2)	−0.0052 (18)	0.0152 (18)	−0.0013 (17)
C17	0.039 (2)	0.025 (2)	0.032 (2)	−0.0077 (19)	0.0208 (19)	−0.0044 (18)
C18	0.036 (2)	0.037 (2)	0.027 (2)	−0.0155 (19)	0.0151 (19)	−0.0079 (19)
C19	0.028 (2)	0.044 (2)	0.026 (2)	−0.0048 (19)	0.0158 (18)	−0.0027 (19)
C20	0.035 (2)	0.031 (2)	0.027 (2)	−0.0017 (19)	0.0176 (18)	−0.0036 (18)
C21	0.031 (2)	0.026 (2)	0.027 (2)	−0.0050 (18)	0.0158 (17)	0.0036 (17)
O22	0.0353 (18)	0.0500 (18)	0.0359 (18)	−0.0150 (16)	0.0139 (15)	−0.0166 (15)
C23	0.050 (3)	0.048 (3)	0.038 (3)	−0.022 (3)	0.023 (3)	−0.017 (2)
O24	0.0267 (16)	0.063 (2)	0.0324 (17)	−0.0006 (15)	0.0134 (14)	−0.0076 (16)
C25	0.029 (2)	0.055 (3)	0.029 (2)	0.005 (2)	0.007 (2)	−0.009 (2)
C26	0.026 (2)	0.051 (3)	0.033 (2)	0.009 (2)	0.005 (2)	−0.010 (2)
O27	0.053 (2)	0.047 (2)	0.0369 (19)	0.0049 (18)	−0.0032 (18)	−0.0113 (16)
O28	0.0387 (19)	0.0484 (19)	0.0340 (18)	0.0061 (16)	0.0068 (15)	−0.0079 (15)
C29	0.043 (3)	0.045 (3)	0.048 (3)	0.015 (2)	0.011 (2)	−0.002 (2)
C30	0.055 (3)	0.069 (3)	0.046 (3)	0.019 (3)	0.024 (3)	0.004 (3)
C31	0.118 (6)	0.066 (4)	0.057 (4)	0.038 (4)	0.036 (4)	0.010 (3)

### Chlorido[4-methoxy-5-(2-oxo-2-propoxyethoxy)-2-(prop-2-en-1-yl)phenyl]\ (quinoline)platinum(II) (III) Geometric parameters (Å, º)

**Table:**

Pt1A—Cl2	2.3281 (14)	C14—C15	1.526 (5)
Pt1A—N3	2.149 (4)	C15—H15A	0.9900
Pt1A—C13	2.091 (4)	C15—H15B	0.9900
Pt1A—C14	2.117 (4)	C15—C16	1.510 (5)
Pt1A—C21	2.003 (4)	C16—C17	1.395 (5)
Pt1B—Cl2	2.205 (6)	C16—C21	1.406 (5)
Pt1B—N3	2.330 (11)	C17—H17	0.9500
Pt1B—C13	2.45 (2)	C17—C18	1.383 (6)
Pt1B—C14	2.178 (9)	C18—C19	1.394 (6)
Pt1B—C21	1.965 (9)	C18—O22	1.382 (5)
N3—C4	1.316 (5)	C19—C20	1.386 (5)
N3—C12	1.354 (5)	C19—O24	1.395 (5)
C4—H4	0.9500	C20—H20	0.9500
C4—C5	1.400 (6)	C20—C21	1.400 (5)
C5—H5	0.9500	O22—C23	1.427 (5)
C5—C6	1.377 (6)	C23—H23A	0.9800
C6—H6	0.9500	C23—H23B	0.9800
C6—C7	1.415 (5)	C23—H23C	0.9800
C7—C8	1.412 (5)	O24—C25	1.425 (5)
C7—C12	1.414 (5)	C25—H25A	0.9900
C8—H8	0.9500	C25—H25B	0.9900
C8—C9	1.350 (6)	C25—C26	1.492 (6)
C9—H9	0.9500	C26—O27	1.197 (5)
C9—C10	1.404 (6)	C26—O28	1.344 (5)
C10—H10	0.9500	O28—C29	1.456 (5)
C10—C11	1.362 (5)	C29—H29A	0.9900
C11—H11	0.9500	C29—H29B	0.9900
C11—C12	1.424 (5)	C29—C30	1.496 (6)
C13—H13C	0.9900	C30—H30A	0.9900
C13—H13D	0.9900	C30—H30B	0.9900
C13—H13A	0.9900	C30—C31	1.524 (7)
C13—H13B	0.9900	C31—H31A	0.9800
C13—C14	1.392 (5)	C31—H31B	0.9800
C14—H14A	1.0000	C31—H31C	0.9800
C14—H14	1.0000		
			
N3—Pt1A—Cl2	85.99 (10)	C13—C14—H14	114.9
C13—Pt1A—Cl2	161.77 (14)	C13—C14—C15	121.7 (4)
C13—Pt1A—N3	91.42 (16)	C15—C14—Pt1A	109.2 (3)
C13—Pt1A—C14	38.62 (14)	C15—C14—Pt1B	101.2 (5)
C14—Pt1A—Cl2	159.56 (15)	C15—C14—H14A	115.7
C14—Pt1A—N3	98.43 (15)	C15—C14—H14	114.9
C21—Pt1A—Cl2	94.43 (13)	C14—C15—H15A	109.7
C21—Pt1A—N3	178.23 (17)	C14—C15—H15B	109.7
C21—Pt1A—C13	87.65 (17)	H15A—C15—H15B	108.2
C21—Pt1A—C14	81.77 (16)	C16—C15—C14	109.7 (3)
Cl2—Pt1B—N3	84.7 (3)	C16—C15—H15A	109.7
Cl2—Pt1B—C13	139.1 (12)	C16—C15—H15B	109.7
N3—Pt1B—C13	78.8 (6)	C17—C16—C15	122.7 (4)
C14—Pt1B—Cl2	173.3 (14)	C17—C16—C21	121.3 (4)
C14—Pt1B—N3	91.5 (4)	C21—C16—C15	115.9 (4)
C14—Pt1B—C13	34.4 (3)	C16—C17—H17	119.8
C21—Pt1B—Cl2	99.5 (3)	C18—C17—C16	120.3 (4)
C21—Pt1B—N3	150.2 (15)	C18—C17—H17	119.8
C21—Pt1B—C13	79.0 (6)	C17—C18—C19	119.1 (4)
C21—Pt1B—C14	81.1 (4)	O22—C18—C17	125.0 (4)
C4—N3—Pt1A	116.6 (3)	O22—C18—C19	115.9 (4)
C4—N3—Pt1B	127.8 (7)	C18—C19—O24	120.2 (4)
C4—N3—C12	118.8 (4)	C20—C19—C18	120.7 (4)
C12—N3—Pt1A	123.8 (3)	C20—C19—O24	118.9 (4)
C12—N3—Pt1B	111.5 (7)	C19—C20—H20	119.4
N3—C4—H4	117.6	C19—C20—C21	121.2 (4)
N3—C4—C5	124.8 (4)	C21—C20—H20	119.4
C5—C4—H4	117.6	C16—C21—Pt1A	116.3 (3)
C4—C5—H5	121.4	C16—C21—Pt1B	113.2 (4)
C6—C5—C4	117.2 (4)	C20—C21—Pt1A	126.3 (3)
C6—C5—H5	121.4	C20—C21—Pt1B	127.8 (4)
C5—C6—H6	120.1	C20—C21—C16	117.3 (4)
C5—C6—C7	119.8 (4)	C18—O22—C23	117.9 (4)
C7—C6—H6	120.1	O22—C23—H23A	109.5
C8—C7—C6	122.3 (4)	O22—C23—H23B	109.5
C8—C7—C12	119.5 (4)	O22—C23—H23C	109.5
C12—C7—C6	118.2 (4)	H23A—C23—H23B	109.5
C7—C8—H8	119.3	H23A—C23—H23C	109.5
C9—C8—C7	121.4 (4)	H23B—C23—H23C	109.5
C9—C8—H8	119.3	C19—O24—C25	114.5 (3)
C8—C9—H9	120.6	O24—C25—H25A	109.2
C8—C9—C10	118.7 (4)	O24—C25—H25B	109.2
C10—C9—H9	120.6	O24—C25—C26	112.2 (4)
C9—C10—H10	118.6	H25A—C25—H25B	107.9
C11—C10—C9	122.8 (4)	C26—C25—H25A	109.2
C11—C10—H10	118.6	C26—C25—H25B	109.2
C10—C11—H11	120.5	O27—C26—C25	127.0 (5)
C10—C11—C12	119.0 (4)	O27—C26—O28	124.3 (5)
C12—C11—H11	120.5	O28—C26—C25	108.7 (4)
N3—C12—C7	121.1 (4)	C26—O28—C29	116.9 (4)
N3—C12—C11	120.3 (4)	O28—C29—H29A	110.3
C7—C12—C11	118.6 (4)	O28—C29—H29B	110.3
Pt1A—C13—H13C	116.4	O28—C29—C30	107.1 (4)
Pt1A—C13—H13D	116.4	H29A—C29—H29B	108.6
Pt1B—C13—H13A	117.6	C30—C29—H29A	110.3
Pt1B—C13—H13B	117.6	C30—C29—H29B	110.3
H13C—C13—H13D	113.4	C29—C30—H30A	109.3
H13A—C13—H13B	114.7	C29—C30—H30B	109.3
C14—C13—Pt1A	71.7 (3)	C29—C30—C31	111.5 (4)
C14—C13—Pt1B	62.0 (5)	H30A—C30—H30B	108.0
C14—C13—H13C	116.4	C31—C30—H30A	109.3
C14—C13—H13D	116.4	C31—C30—H30B	109.3
C14—C13—H13A	117.6	C30—C31—H31A	109.5
C14—C13—H13B	117.6	C30—C31—H31B	109.5
Pt1A—C14—H14A	115.7	C30—C31—H31C	109.5
Pt1B—C14—H14	114.9	H31A—C31—H31B	109.5
C13—C14—Pt1A	69.7 (2)	H31A—C31—H31C	109.5
C13—C14—Pt1B	83.7 (8)	H31B—C31—H31C	109.5
C13—C14—H14A	115.7		
			
Pt1A—N3—C4—C5	167.9 (3)	C15—C16—C17—C18	174.9 (4)
Pt1A—N3—C12—C7	−168.1 (3)	C15—C16—C21—Pt1A	5.0 (5)
Pt1A—N3—C12—C11	11.9 (5)	C15—C16—C21—Pt1B	−11.5 (9)
Pt1A—C13—C14—C15	100.5 (4)	C15—C16—C21—C20	−177.8 (3)
Pt1A—C14—C15—C16	28.6 (4)	C16—C17—C18—C19	3.2 (6)
Pt1B—N3—C4—C5	160.7 (5)	C16—C17—C18—O22	−175.2 (4)
Pt1B—N3—C12—C7	−164.3 (4)	C17—C16—C21—Pt1A	−179.2 (3)
Pt1B—N3—C12—C11	15.7 (5)	C17—C16—C21—Pt1B	164.3 (9)
Pt1B—C13—C14—C15	99.3 (4)	C17—C16—C21—C20	−2.0 (6)
Pt1B—C14—C15—C16	40.6 (8)	C17—C18—C19—C20	−3.2 (6)
N3—C4—C5—C6	1.0 (7)	C17—C18—C19—O24	−177.7 (4)
C4—N3—C12—C7	1.1 (6)	C17—C18—O22—C23	−3.9 (6)
C4—N3—C12—C11	−178.9 (4)	C18—C19—C20—C21	0.5 (6)
C4—C5—C6—C7	1.1 (6)	C18—C19—O24—C25	−69.9 (5)
C5—C6—C7—C8	178.0 (4)	C19—C18—O22—C23	177.7 (4)
C5—C6—C7—C12	−2.0 (6)	C19—C20—C21—Pt1A	178.9 (3)
C6—C7—C8—C9	−179.1 (4)	C19—C20—C21—Pt1B	−162.0 (11)
C6—C7—C12—N3	0.9 (6)	C19—C20—C21—C16	2.0 (6)
C6—C7—C12—C11	−179.1 (4)	C19—O24—C25—C26	−86.2 (4)
C7—C8—C9—C10	−1.7 (7)	C20—C19—O24—C25	115.6 (4)
C8—C7—C12—N3	−179.1 (4)	C21—C16—C17—C18	−0.6 (6)
C8—C7—C12—C11	0.9 (6)	O22—C18—C19—C20	175.4 (4)
C8—C9—C10—C11	0.7 (7)	O22—C18—C19—O24	0.9 (6)
C9—C10—C11—C12	1.1 (7)	O24—C19—C20—C21	175.1 (3)
C10—C11—C12—N3	178.1 (4)	O24—C25—C26—O27	4.0 (7)
C10—C11—C12—C7	−1.8 (6)	O24—C25—C26—O28	−175.4 (3)
C12—N3—C4—C5	−2.1 (7)	C25—C26—O28—C29	179.3 (4)
C12—C7—C8—C9	0.9 (6)	C26—O28—C29—C30	−179.8 (4)
C13—C14—C15—C16	−48.9 (5)	O27—C26—O28—C29	−0.1 (7)
C14—C15—C16—C17	161.5 (4)	O28—C29—C30—C31	177.4 (4)
C14—C15—C16—C21	−22.8 (5)		

### Chlorido[4-methoxy-5-(2-oxo-2-propoxyethoxy)-2-(prop-2-en-1-yl)phenyl]\ (quinoline)platinum(II) (III) Hydrogen-bond geometry (Å, º)

*Cg*1 is the centroid of ring C16–C21.

**Table:**

*D*—H···*A*	*D*—H	H···*A*	*D*···*A*	*D*—H···*A*
C9—H9···O27^i^	0.95	2.59	3.445 (5)	150
C23—H23*C*···Cl2^ii^	0.98	2.70	3.618 (6)	157
C29—H29*B*···O24^iii^	0.99	2.50	3.381 (7)	148
C6—H6···*Cg*1^iv^	0.95	2.73	3.269 (5)	117

Symmetry codes: (i) −*x*+1, *y*−1/2, −*z*−1/2; (ii) *x*, −*y*+3/2, *z*−1/2; (iii) −*x*, *y*+1/2, −*z*−1/2; (iv) −*x*+1, *y*+1/2, −*z*+1/2.
